# Phototherapy techniques for the management of musculoskeletal disorders: strategies and recent advances

**DOI:** 10.1186/s40824-023-00458-8

**Published:** 2023-11-28

**Authors:** Zhenhe Zhang, Rong Wang, Hang Xue, Samuel Knoedler, Yongtao Geng, Yuheng Liao, Michael Alfertshofer, Adriana C. Panayi, Jie Ming, Bobin Mi, Guohui Liu

**Affiliations:** 1 Department of Orthopedics, Union Hospital, Tongji Medical College, Huazhong University of Science and Technology, 1277 Jiefang Avenue, Wuhan, 430022 China; 2 Hubei Province Key Laboratory of Oral and Maxillofacial Development and Regeneration, Wuhan, 430022 China; 3 Department of Breast and Thyroid Surgery, Union Hospital, Tongji Medical College, Huazhong University of Science and Technology, 1277 Jiefang Avenue, Wuhan, 430022 China; 4 Division of Plastic Surgery, Brigham and Women’s Hospital, Harvard Medical School, Boston, MA 02152 USA; 5 Institute of Regenerative Biology and Medicine, Helmholtz Zentrum München, Max-Lebsche-Platz 31, 81377 Munich, Germany; 6 Division of Hand, Plastic and Aesthetic Surgery, Ludwig-Maximilians-University Munich, Munich, Germany; 7 Department of Hand, Plastic and Reconstructive Surgery, Microsurgery, Burn Center, BG Trauma Center Ludwigshafen, University of Heidelberg, Ludwig-Guttmann-Strasse 13, 67071 Ludwigshafen, Rhine Germany

**Keywords:** Photothermal therapy, Photodynamic therapy, Bone regeneration, Bone infection, Bone tumor, Osteoarthritis, Rheumatoid arthritis

## Abstract

Musculoskeletal disorders (MSDs), which include a range of pathologies affecting bones, cartilage, muscles, tendons, and ligaments, account for a significant portion of the global burden of disease. While pharmaceutical and surgical interventions represent conventional approaches for treating MSDs, their efficacy is constrained and frequently accompanied by adverse reactions. Considering the rising incidence of MSDs, there is an urgent demand for effective treatment modalities to alter the current landscape. Phototherapy, as a controllable and non-invasive technique, has been shown to directly regulate bone, cartilage, and muscle regeneration by modulating cellular behavior. Moreover, phototherapy presents controlled ablation of tumor cells, bacteria, and aberrantly activated inflammatory cells, demonstrating therapeutic potential in conditions such as bone tumors, bone infection, and arthritis. By constructing light-responsive nanosystems, controlled drug delivery can be achieved to enable precise treatment of MSDs. Notably, various phototherapy nanoplatforms with integrated imaging capabilities have been utilized for early diagnosis, guided therapy, and prognostic assessment of MSDs, further improving the management of these disorders. This review provides a comprehensive overview of the strategies and recent advances in the application of phototherapy for the treatment of MSDs, discusses the challenges and prospects of phototherapy, and aims to promote further research and application of phototherapy techniques.

**Graphical Abstract:**

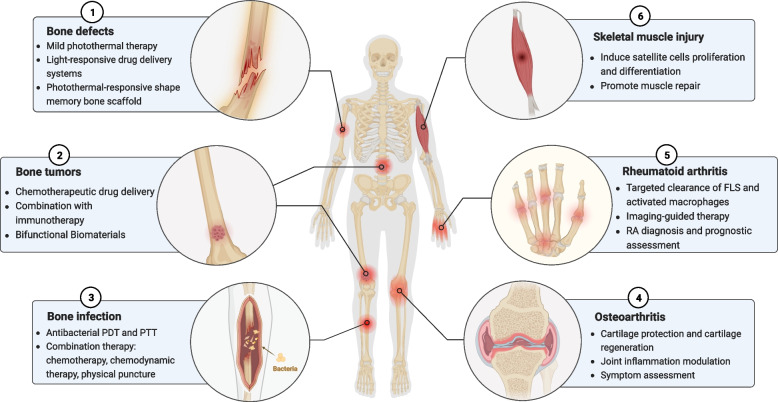

## Introduction

The musculoskeletal system comprises the bones, cartilage, muscles, tendons, and ligaments, playing a critical role in providing structural support and coordinating movements within the human body. This complex system possesses intrinsic regenerative capabilities, however, its reparative capacity is subject to inherent limitations, especially concerning the regeneration of cartilage, tendons, and ligaments [1]. Pathological conditions, including trauma, infections, autoimmune diseases, and the physiological aging process, can induce irreversible damage to the musculoskeletal system, thus culminating in musculoskeletal disorders (MSDs) [2, 3]. These disorders may manifest as chronic pain and functional impairment, affecting both physical and psychological well-being, impairing work capability, and, in severe cases, even leading to disability or death. A recent epidemiological study indicates that over 1.3 billion people worldwide are affected by MSDs, and the prevalence of MSDs is expected to rise as the population ages, placing an enormous burden on the healthcare system [4].

The treatment of MSDs has always been a clinical challenge. In cases of severe tissue defects, such as bone and cartilage defects, surgery is often inevitable. Albeit autografts, allografts, and xenografts pose essential treatment options for these diseases, their use is limited due to issues like scarcity, site morbidity, pain, and risk of immunogenicity [3]. Bone tissue engineering scaffolds show promise as an alternative to tissue transplantation by mimicking the extracellular matrix, supporting cell growth, and facilitating tissue regeneration through drug delivery [5]. However, the limited functionality of scaffolds and uncontrolled drug release hinder the wider application. For diseases involving abnormally proliferating or activated cells, such as bone tumors and rheumatoid arthritis (RA), current treatment modalities involve surgery or chemotherapy [6, 7]. However, surgery is invasive and chemotherapeutic drugs can have potentially harmful side effects. Several drug delivery nanosystems with high specific areas and controlled structures have garnered attention for targeting disease sites through appropriate modifications and inducing abnormal cell death through drug release [8]. Nevertheless, the effectiveness of these drug delivery nanosystems relies on conventional chemotherapeutic drugs, which show limitations in overcoming the potentially harmful side effects of chemotherapeutic drugs, necessitating further improvement in therapeutic outcomes.

Phototherapy, as a controlled and non-invasive technique, has received significant attention as a potential treatment for various diseases. Photothermal therapy (PTT) relies on photothermal agents to convert light energy into heat, while photodynamic therapy (PDT) involves photosensitizers generating reactive oxygen species (ROS) upon light irradiation [9]. PTT allows for controlled thermal therapy, while the ROS generated by PDT has a limited diffusion distance and short lifetime [10]. By designing PTT and PDT platforms with targeted effects, precise and efficient abnormal cell-killing effects can be achieved with minimized damage to surrounding healthy tissues, making phototherapy a promising approach for treating tumors and infections [11]. In addition, light-responsive drug delivery systems incorporating photothermal agents into drug carriers enable controlled drug release via structural changes of the drug carrier upon laser irradiation. This technology can be also utilized to deliver antibacterial agents for treating infections, and growth factors for bone repair [12]. Furthermore, advances in nanotechnology have led to the development of multifunctional nanomaterials that combine PTT and PDT with other therapeutic modalities, such as chemotherapy, chemodynamic therapy (CDT), and gene therapy, which improves therapeutic efficacy [13]. Recent advancements in photoacoustic imaging (PAI), fluorescence imaging, and near-infrared (NIR) imaging technologies have facilitated the design of nanomaterials with integrated phototherapy and imaging capabilities, enabling early diseases diagnosis, prognosis, and guided therapy through in vivo nanomedicine distribution [14].

**Fig. 1:**
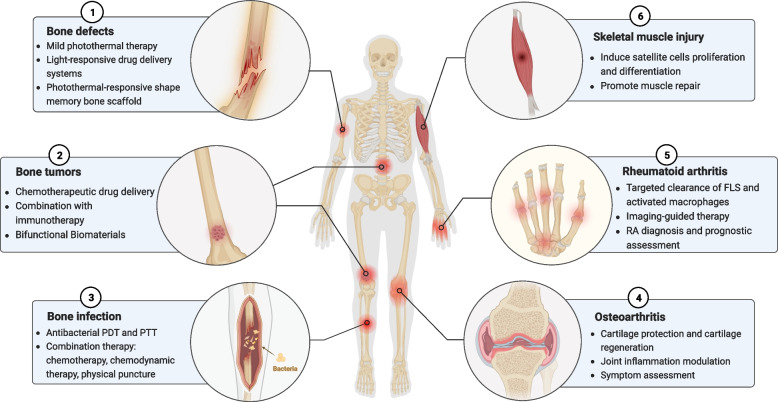
Phototherapy techniques for the management of musculoskeletal disorders. (FLS, Fibroblast-like synoviocytes)

Over the past five years, the application of phototherapy in MSDs has been gaining increasing attention. Researchers have developed multifunctional nanomaterials and scaffolds, proposing various strategies for bone defects, bone infections, bone tumors, osteoarthritis (OA), RA, and skeletal muscle injuries. This review summarizes the progress of phototherapy treatment strategies and applications (Fig. 1). Further, we discuss the challenges and prospects associated with the implementation of phototherapy in MSDs, with the aim of encouraging further research and application of phototherapy in treating these complex disorders.

## Bone Defects

Bone integrity can be compromised by various factors, including trauma, metabolic diseases, and malignant tumors [15]. While bone tissues possess a natural ability to repair themselves, critical-size bone defects, chronic diseases, infections, and inadequate fixation often result in delayed healing or non-healing of the bone defects [16]. To address this challenge, bone tissue engineering scaffolds, including metal scaffolds, polymer scaffolds, and hydrogels, have emerged as promising alternatives to traditional bone grafting methods. These scaffolds are characterized by modifiable structures, excellent mechanical properties, drug-loading capacity, and high bioactivity, rendering them ideal candidates for treating bone defects [17].

More recently, significant attention has been directed towards bone tissue engineering scaffolds that can be utilized in PTT of bone repair. These scaffolds attain the ability to be used for PTT by selecting appropriate materials or incorporating photothermal agents [18]. Herein, we provide a comprehensive overview of the advancements in light-responsive materials within the field of bone repair.

### Light propagation and phototherapy for bone regeneration

Phototherapy for bone regeneration is usually achieved by laser irradiation of light-responsive bone scaffolds in the body, which means that the light penetrates the tissue and still has sufficient light energy and light range [19]. However, light inevitably interacts with tissues (mainly scattering and absorption) as it propagates through them. Therefore, understanding the propagation characteristics of light in tissues and selecting light in a specific wavelength range with low levels of scattering and absorption is essential for phototherapy of bone.

In bone phototherapy, most studies place bone scaffolds at the site of bone defect, and the light penetration ability is mainly affected by soft tissue. Due to the inhomogeneity of the refractive index in the tissue, incident light entering the tissue is scattered, causing the light to deviate from its original path, resulting in light attenuation [20]. By analyzing the light scattering characteristics of skin, subcutaneous tissue and muscle, it was found that the light scattering ability of skin was significantly stronger than that of subcutaneous tissue and muscle tissue in the range of 400-700 nm [21, 22]. It has been reported that 408 nm visible light can only penetrate 1 mm into the skin, and 633 nm light has a penetration depth of only 6.3 mm, suggesting that the skin significantly restricts the entry of visible light into the depths of the body [23]. It is noteworthy that the light scattering coefficients of skin, subcutaneous tissue, and muscle show a significant decrease in the wavelength range of 400-1700 nm, which is especially pronounced in the 400-700 nm range [21]. When the wavelength is greater than 700nm, the light scattering coefficients of these tissues tend to stabilize, which suggests that using light with wavelengths greater than 700 nm can facilitate the penetration of soft tissue to reach deep-seated bones for phototherapy. In addition to light scattering, light absorption likewise causes energy attenuation during the propagation of light in tissues, affecting phototherapy for bone regeneration [19]. Water and blood (rich in hemoglobin and water) are thought to dominate light absorption in tissues, while some other components, such as melanin and fat, also absorb light that penetrates into tissues [21]. Light is also converted into heat when absorbed by these components, which means there is a risk of overheating and damaging healthy tissue. However, each of these tissue components has a unique spectral distribution, for example, blood primarily absorbs light at wavelengths less than 600 nm [22]. Additionally, water exhibits local absorption peaks at around 970 nm, 1200 nm, and 1450 nm, while showing a local minimum at 808 nm [24, 25]. It is due to these special tissue optical properties that light can be minimized from absorption and scattering within some specific windows [26]. Currently, there are confirmed biological transparency windows, including the first window from 700 nm to 950 nm (NIR-I), the second window covering the region from 1000 to 1350 nm (NIR-II), and the third window from 1550 to 1870 nm (NIR-III) [27]. Within these windows, light can penetrate several centimeters of tissue, which is beneficial for achieving phototherapy for bone repair by allowing light to penetrate soft tissue.

Bone is characterized by high mineralization, rich collagen content, relatively low lipid content, and the presence of highly pigmented blood cells in the bone marrow space [28]. For light-responsive bone implants placed within the bone marrow cavity, achieving phototherapy requires light to first penetrate cortical bone, trabecular bone, and bone marrow. Studies of the optical properties of bone tissue have shown that light absorption by bone comes mainly from the water and hemoglobin in it, while the solid bone components are involved in absorbing only small amounts of light [29]. Currently, light scattering is considered the primary obstacle affecting light penetration through bone [19, 30]. It is worth noting that, like other soft tissues, the light scattering coefficient of bone decreases as the wavelength of light increases [29]. In particular, light within the wavelength range of 700 nm to 1700 nm exhibits similar light scattering coefficients in both bone tissue and skin tissue [21]. Therefore, it is reasonable to believe that light with wavelengths located in the biological transparency windows can better promote phototherapy for bone marrow cavity implants. In addition, bone repair is a dynamic process and the composition of the surface of the bone scaffold can change significantly leading to different optical properties, e.g., hematomas in the early stages of bone injury may result in stronger light absorption, whereas the formation of a hard bone scab may result in stronger light scattering [21, 31]. This dynamic bone repair process may lead to variations in the light penetration capability in different stages of bone healing. However, this potential impact still requires further research.

### Mechanism of mild PTT to accelerate bone regeneration

**Fig. 2:**
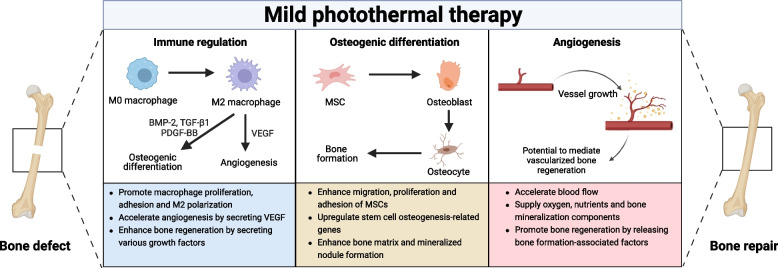
Mechanism of mild PTT regulating bone regeneration. (BMP-2, Bone morphogenetic protein-2; TGF-β1, Transforming growth factor-β1; PDGF-BB, Platelet-derived growth factor-BB; VEGF, Vascular endothelial growth factor; MSC, Mesenchymal stem cell.)

PTT has been shown to regulate cellular function. Here, the mechanism by which mild PTT directly regulates bone regeneration was summarized (Fig. 2). Mesenchymal stem cells (MSCs) play a crucial role in bone repair by migrating to the site of bone defects, proliferating, and differentiating into osteoblasts and osteocytes to form mineralized bone [32]. Recent studies have shown that mild PTT directly promotes the proliferation of MSCs and osteoblasts [33, 34]. Tong et al. found that MSCs subjected to 7 days of mild PTT at 40.5°C exhibited minimal apoptosis and necrosis, with significantly higher cell viability compared to the non-photothermal stimulated group, indicating that mild PTT enhances MSCs survival and proliferation [35]. In another study, mild PTT was found to increase the viability of MC3T3-E1 osteoblast-like cells by 87%, surpassing the pro-proliferative efficiency of silicon and phosphorus ions [12]. Further, mild PTT alters the cytoskeleton of MSCs and osteoblasts, resulting in enhanced adhesion and migration [36]. Zhang et al. revealed that after photothermal stimulation at 42°C, the cytoskeleton of MSCs exhibited a flatter shape and larger diffusion area, suggesting a potential contribution of mild PTT to the adhesion of MSCs [37]. Similarly, it was shown in other studies that MC3T3-E1 cells on the surface of the material expressed more linear filamentous pseudopods and exhibited better adhesion and spreading ability after mild photothermal therapy [12, 38]. Further studies also revealed that mild photothermal therapy stimulated osteogenic differentiation. Specifically, after mild photothermal stimulation, MSCs and MC3T3-E1 cells showed upregulation of osteogenic-related genes, increased ALP activity, and more calcium salt deposition, indicating enhanced osteogenic differentiation [12, 39]. Overall, these findings suggest that mild PTT promotes the proliferation, adhesion, and migration of MSCs and osteoblasts, as well as the formation of bone matrix and mineralized nodules, thereby contributing to bone repair.

While the beneficial effects of mild PTT have been observed, the underlying molecular mechanisms involved are not yet fully understood [40]. The mammalian heat shock protein (HSP) family is involved in regulating various cellular processes. For example, HSP47 regulates the cross-linking of collagen to produce the extracellular matrix, while HSP27, HSP70, and HSP90 contribute to the folding of newly synthesized or misfolded proteins, thereby regulating cell proliferation and differentiation [41–43]. Recent evidence has pointed to an association between the promotion of stem cells proliferation, differentiation and osteoblast maturation by mild PTT with upregulation of HSP family protein expression and its downstream pathways [44, 45]. For example, Ma et al. noticed upregulation of HSP40 and HSP70 expression in MSCs upon mild PTT stimulation at 40.5°C [39]. Qu et al. noted that mild PTT was able to upregulate HSP70 in adipose-derived stem cells and further promoted extracellular signal-regulated kinase (ERK) phosphorylation, which upregulated osteogenesis-related marker genes bone sialoprotein and runt-related transcription factor-2 (Runx-2) expression [46]. Additionally, mild PTT was found to stimulate the expression of HSP27 and HSP90 in osteoblasts, leading to activation of the p38/Smad and ERK pathways, respectively, and the upregulation of osteogenesis-related marker genes, including alkaline phosphatase (ALP), Runx-2, bone morphogenetic protein (BMP)-2, osteopontin (OPN), osteocalcin (OCN) and collagen I (Col-I) [12]. Transcriptome sequencing studies have revealed the involvement of pathways such as PI3K/Akt pathway, β-catenin, MAPK, Rap1 and Smad in mild PTT-induced MSCs differentiation [34, 40]. Overall, the available studies suggest that mild PTT may be involved in the regulation of multiple pathways in stem cells to promote osteogenic differentiation, and these involved pathways need to be confirmed by more experiments.

The immune response also plays a pivotal role in bone repair. In the initial stages of bone repair, the site of bone defects is primarily infiltrated by M1-type macrophages, which aid in the removal of necrotic tissue and combat infection by promoting inflammatory responses [47]. As inflammation progresses, macrophages gradually transition to the M2 phenotype, influencing MSCs osteogenic differentiation through the secretion of growth factors. However, in the presence of unfavorable factors such as infection or diabetes mellitus, M1-type macrophages may accumulate in large numbers at the site of bone defects for prolonged periods of time, resulting in delayed bone repair [48]. Recent studies have revealed that mild PTT not only affects MSCs behavior but also mediates bone repair by regulating immune homeostasis. Li et al. demonstrated that periodic mild photothermal stimulation at 41°C promoted macrophage proliferation, adhesion, and M2 polarization [49]. This acceleration of bone formation occurred through the secretion of growth factors, such as platelet-derived growth factor (PDGF)-BB, transforming growth factor (TGF)-β1, and BMP-2, which recruit MSCs and promote osteogenic differentiation and ECM mineralization. Transcriptomic analysis further indicated that mild PTT promoted macrophage M2 polarization via activation of the PI3K-AKT1 signaling pathway. In another study, mild PTT at 42°C induced macrophage M2 polarization, leading to accelerated angiogenesis and bone formation. This effect was achieved through the release of vascular endothelial growth factor (VEGF) and BMP-2 from M2-type macrophages, respectively. As a result, mild PTT facilitated the healing of critical-size cranial defects in rats [38].

Blood vessels are essential for bone repair as they provide essential oxygen, nutrients, and bone mineralization components (such as calcium and phosphate) [50]. Accelerating local blood flow through PTT could be an effective approach to enhance the supply of these substances, thereby accelerating bone repair [51, 52]. Moreover, recent research by Qu et al. found that mild PTT from MXene (Ti_3_C_2_T_x_) nanosheets not only modulated MSCs osteogenic differentiation but also promoted endothelial cell migration and angiogenesis [46]. Although the study did not directly demonstrate angiogenesis-mediated bone repair, it is reasonable to believe that mild PTT has the potential to promote vascularized bone regeneration, considering that vascular endothelial cells also contribute to bone repair through the production of osteoclastogenic factors [50]. However, further investigations are needed to confirm this potential.

While mild PTT has shown promising effects on MSCs osteogenic differentiation and macrophage polarization to enhance bone repair, the exact temperature for its optimal efficacy remains inconclusive. Previously, it was commonly believed that thermal stimulation above 43 °C resulted in cell death, hence, mild PTT for induction of bone regeneration was controlled within the range of 40-43 °C [39, 46, 53]. However, recent studies have demonstrated that photothermal stimulation at 44°C-46°C also promotes MSCs osteogenic differentiation and accelerates bone repair in vivo [40, 52, 54]. In addition, macrophages release more pro-inflammatory factors after the initial mild photothermal stimulation and they exhibit anti-inflammatory phenotypes only after subsequent cycles of mild photothermal stimulation [49]. Since the material properties of the photothermal material itself (e.g., roughness, morphology, hydrophilicity), as well as the released drug and the mode of photothermal stimulation (including temperature, duration, and period) can impact cellular behaviors, it is essential to conduct systematic investigations to determine the most suitable photothermal stimulation mode [12, 55]. These studies will ultimately help optimize the therapeutic potential of mild PTT for bone repair applications.

### Light-responsive drug delivery systems

Photothermal agents offer promising applications in constructing light-responsive drug delivery systems for the controlled release of small molecule drugs, growth factors, and inorganic ions, making them valuable in bone tissue engineering prospects [52]. One commonly employed strategy involves light-responsive drug delivery to directly modulate MSCs or osteoblast function. For instance, Xue et al. utilized the micro arc oxidation technique to create silicon and phosphorus doped functionalized coatings on titanium (Ti) surface [12]. NIR irradiation accelerated the release of silicon and phosphorus ions, synergizing with mild PTT to promote osteoblast differentiation and expedite osseointegration. Similarly, Liu et al. developed a thermosensitive hydrogel-coated PTHrP-2 (a novel parathyroid hormone-related peptide)-loaded mesoporous bioactive glass (MBG) [56]. Under NIR irradiation, the thermosensitive hydrogel acted as a gate for MBG pores, contracting due to a phase transition at elevated temperatures, thereby opening the mesopores of MBG to promote PTHrP-2 release. This accelerated MSCs osteogenic differentiation and bone repair process.

**Fig. 3:**
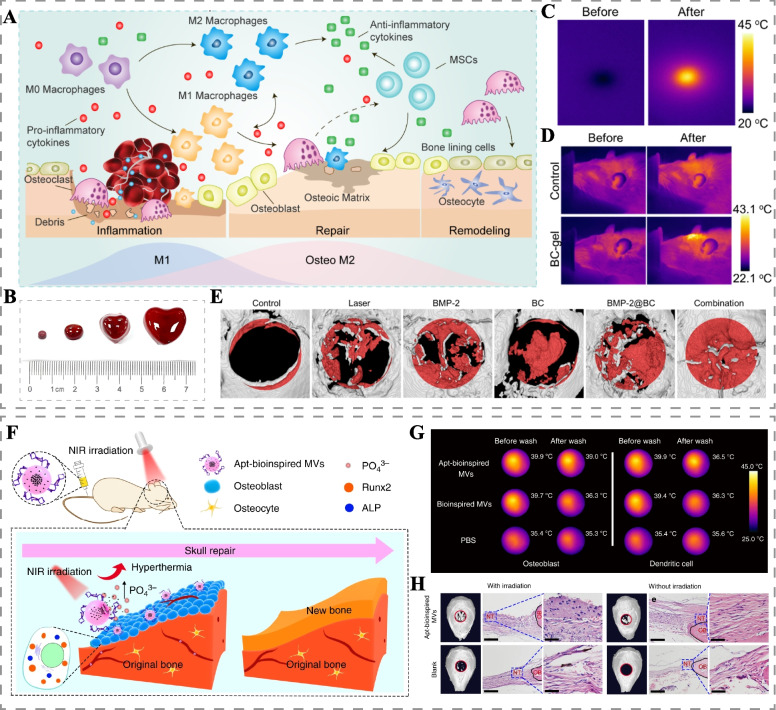
Light-responsive drug delivery systems for bone repair. **A** Schematic of light-responsive BMP-2@BC hydrogel regulating bone repair. **B** BMP-2@BC hydrogels molded into different shapes. Infrared thermal image of BMP-2@BC hydrogel in vitro **C** and in vivo **D**. **E** Micro-CT 3D reconstruction of the skull after 8 weeks of treatment. Reproduced with permission [45]. Copyright 2021, Elsevier. **F** Schematic illustration of Apt-bioinspired MVs promoting skull defect repair. **G** Infrared thermal images of osteoblasts and dendritic cells incubated with bioinspired MVs and Apt-bioinspired MVs before and after washing. (For detection of osteoblast targeting ability of Apt-bioinspired MVs.) **H** Micro-CT 3D reconstruction and H&E-staining images of skull defects in treated mice. Scale bars of H&E-staining images:100 μm (left) and 50 μm (right). Reproduced with permission [59]. Copyright 2019, Springer Nature

Bone repair is a complex process involving immunity, blood vessels, and nerves. Employing light-responsive drug delivery to modulate the immune microenvironment, angiogenesis, and neural regeneration is a significant strategy for promoting bone repair [56, 57]. Li et al. developed a bilayer polycaprolactone (PCL) nanofiber bionic periosteum doped with Nd@WH (Nd, the trivalent form of neodymium element; WH, whitlockite) nanoparticles, which promoted nerve and blood vessel regeneration by releasing Mg^2+^ and synergistically accelerated the bone defect repair by PTT and Ca^2+^ and PO4^3-^ release [57]. Recently, Fan et al. designed a NIR-responsive BMP-2 delivery system using blood clots as carriers (BMP-2@BC hydrogel) [45]. The blood clots themselves carried various growth factors that recruited more macrophages and promoted macrophage M1 polarization in the early stages of bone repair while inducing more M2-type macrophage formation in the later stages (Fig. 3A). Additionally, the crimson blood clots achieved photothermal conversion, accelerating the release of growth factors and BMP-2 in a NIR-responsive manner, synergizing with mild PTT and accelerating the healing of critical cranial defects in rats (Fig. 3B-E). However, it is crucial to note that excessive temperature during PTT may be detrimental to the growth factors loaded in drug delivery systems. A recent study found that osteogenic peptides exposed to 47°C photothermal therapy for 10 minutes reduced their ability to promote osteogenic differentiation of MSCs, highlighting the need for careful control of photothermal temperature when using growth factor-loaded materials [58].

Black phosphorus (BP), a novel photothermal agent composed of phosphorus monomers, is currently the most commonly used photothermal material for the treatment of bone defects [35, 60]. Scaffolds containing BP can be employed to construct self-produced phosphates photothermal systems, as BP decomposes into phosphates in the presence of oxygen and water [61]. Consequently, these phosphates can bind to local Ca^2+^ to achieve bone mineralization. Recently, Wang et al. combined SrCl_2_ and BP nanosheets into poly (lactic acid-glycolic acid copolymer) (PLGA) microspheres (BP-SrCl_2_/PLGA) for bone regeneration [60]. Under NIR irradiation, BP underwent photothermal conversion and heat up. Once the temperature exceeded 45 °C, the PLGA shell was destroyed, promoting the release of Sr^2+^ and the supply of phosphates from the BP, thereby accelerating the repair of femoral head defects in rats. In another study, BP quantum dots (BPQDs) were encapsulated in PLGA nanoparticles and grafted with osteoblast-specific aptamer to obtain Apt-bioinspired MVs [59]. Apt-bioinspired MVs specifically targeted osteoblasts, promoted their differentiation and biomineralization through mild PTT and released phosphates under NIR irradiation, thereby facilitating the healing of skull defects in mice (Fig. 3F-H).

### Photothermal-responsive shape memory bone scaffold

**Fig. 4:**
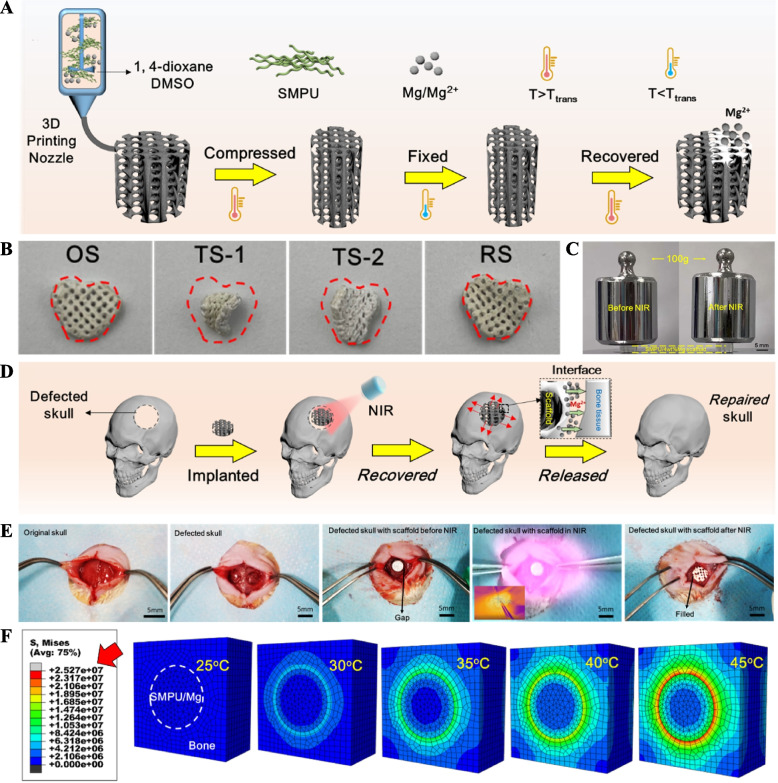
Photothermal-responsive SMPU/Mg scaffolds for bone repair. **A** Schematic diagram of preparation of small volume SMPU/Mg scaffolds and recovery of their shape. **B** The process of shape recovery of SMPU/Mg scaffolds under NIR (808 nm, 1 W/cm^2^) irradiation. OS, original shape; TS-1, Temporary shape-1; TS-2, Temporary shape-2; RS, recovered shape. **C** SMPU/Mg scaffold shape recovery induced by NIR laser under 100g weight compression. Scale bar: 50 μm. **D** Schematic illustration of in vivo application of SMPU/Mg scaffolds. **E** The process of implantation of SMPU/Mg scaffolds and shape recovery under NIR irradiation. Scale bar: 5mm. **F** Equivalent stress distribution by finite element analysis of SMPU/Mg scaffolds indicate tight contact of the scaffold with the skull. Reproduced with permission [54]. Copyright 2022, Elsevier

Shape memory polymers (SMPs) represent a class of materials capable of altering their shape and size in response to various stimuli, including heat, pH, water, light, and magnetism [62]. Integrating photothermal agents into thermal-responsive shape memory polymers enables shape recovery induced by NIR irradiation [63]. Such photothermal-responsive shape memory bone scaffolds can be molded into small sizes in vitro and induced to recover their shape in vivo using NIR irradiation [64]. Recently, Zhang et al. successfully doped magnesium, a photothermal agent, into thermal-responsive shape memory polyurethane (SMPU) and fabricated SMPU/Mg scaffolds using the low temperature rapid prototyping (LT-RP) three-dimensional (3D) printing technique [54]. By applying external force to compress the SMPU/Mg scaffold above the transition temperature and temporarily fixing the shape below the transition temperature, they obtained small volumes of SMPU/Mg scaffold, which allowed the scaffold to be implanted into cranial defects with minimal wounds (Fig. 4A). Under NIR irradiation, the scaffolds gradually warmed up due to the photothermal effect, recovering their original shape to match the shape of the bone defect when the temperature exceeded the transition temperature (Fig. 4B-E). This process accelerated cranial defect repair through the release of Mg^2+^ (Fig. 4F). In another study, Wang et al. employed cryogenic four-dimensional (4D) printing to create a hierarchical porous nanocomposite scaffold comprising BP nanosheets, osteogenic peptides, and β-tricalcium phosphate/poly (lactic acid-co-trimethylene carbonate) for irregular bone defects based on computed tomography (CT) scan data [58]. Under photothermal conditions at 45°C, the scaffold’s shape was restored to match the irregular bone defects, facilitating bone repair through the release of osteogenic peptides.

Despite the promising potential of photothermal-responsive shape memory polymer scaffolds for bone repair applications, several considerations must be addressed in future studies. First, selecting an appropriate transformation temperature is pivotal. Transition temperatures below body temperature may cause the materials to recover their shape prematurely during implantation, whereas excessively high transition temperatures could lead to tissue damage around the scaffolds or inactivation of growth factors loaded within the materials. In addition, many shape memory polymers exhibit limited degradation ability, necessitating research efforts to adapt material degradation to the physiological process of bone repair. This can be achieved by carefully choosing suitable SMP materials or modifying existing SMP materials to enhance their degradation properties.

## Bone Infection

**Fig. 5:**
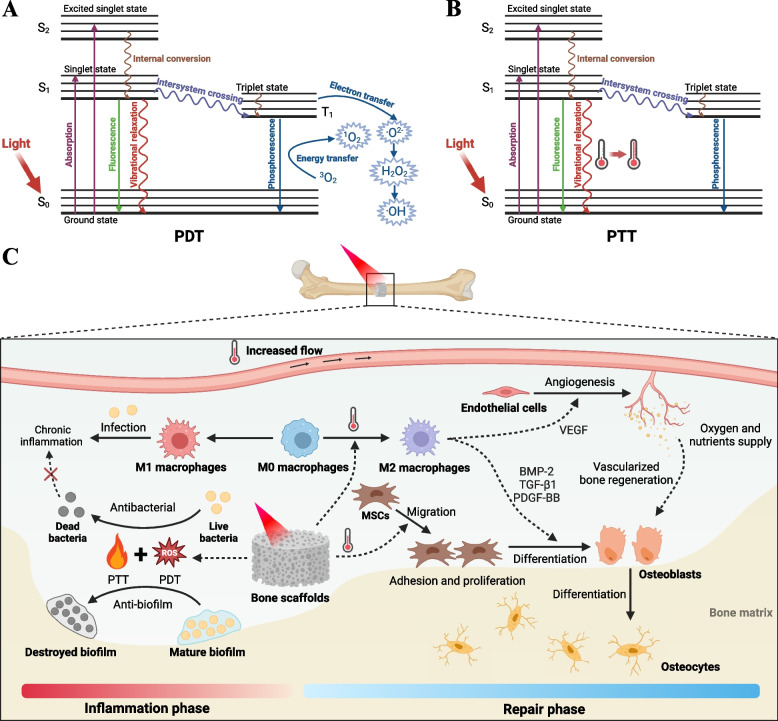
Phototherapy techniques for the treatment of bone infections. Jablonski diagrams of the photophysical and photochemical basis of PDT **A** and PTT **B**. **C** Mechanisms of PTT- and PDT-based techniques for the treatment of infected bone defects

Open bone injuries and the application of orthopedic implants pose an increased risk of bacterial invasion and colonization [65]. Upon infection of the bone, bacteria stimulate inflammatory cells to secrete various inflammatory factors, upregulate osteoblast RANKL/OPG ratios, promote osteoclast formation, and induce osteoclast apoptosis, resulting in an imbalance between bone resorption and bone formation and impaired bone repair [66]. Globally, the reported annual incidence of osteomyelitis is 21.8 out of hundred thousand people, and this figure continues to rise each year [67]. Unfortunately, due to specific pathogenic factors such as intracellular infection, osteocyte lacuno-canalicular network (OLCN) invasion, biofilm formation, and abscess formation, eradicating bacteria often proves challenging, leading to recurrence of bone infection in approximately 17% of patients [68, 69]. PDT and PTT, as non-contact, broad-spectrum and highly effective anti-infection techniques, have shown great potential in the treatment of bone infections. Here, we summarize the mechanisms associated with PDT- and PTT-based phototherapy techniques in the treatment of infected bone defects (Fig. 5).

### Antibacterial PDT and PTT

Antibacterial PDT is mainly mediated by the generation of hydroxyl radicals (•OH), singlet oxygen (^1^O_2_) and superoxide anions (O_2_^•−^) catalyzed by photosensitizers upon light excitation. These ROS induce oxidative damage to polysaccharides, lipids, proteins, and nucleic acids, leading to cell membrane disruption, DNA damage, and enzyme inactivation, ultimately causing bacterial death [9]. The multifaceted antibacterial mechanisms of ROS confer PDT with a broader antibacterial spectrum and reduced susceptibility to resistance compared to antibiotics, making it highly effective in the treatment of bone infections. In one study, the photosensitizer toluidine blue was used in PDT for osteomyelitis in rats, resulting in more than 99% reduction in bacterial load after 30 days of treatment, without bone resorption or microabscesses observed [70].

In addition to provide a favorable anti-infection microenvironment, bone repair can also be actively promoted by incorporating osteogenic drugs [71]. Yuan et al. introduced carbon-ZnO into PLLA, imparting the scaffold not only with excellent antibacterial properties via PDT but also promoting the formation of mineralized nodules in human osteoblast-like MG-63 cells through the release of Zn^2+^ [72]. Recently, Xie et al. designed a self-assembled bilayer hydrogel with spatiotemporal modulation of the immune microenvironment for osteomyelitis treatment [48]. The upper layer of this hydrogel was an AC_10_A hydrogel loaded with Ag_2_S QDs@DSPE-mPEG_2000_-Ce6/Aptamer (AD-Ce6/Apt), while the lower layer was an AC_10_ARGD hydrogel loaded with MSCs. After implantation, the upper hydrogel first released AD-Ce6/Apt, which exhibited direct antibacterial effects via ROS generated by PDT and enhanced the antibacterial effect by recruiting and inducing macrophage M1 polarization via the generated ROS in the first 3-4 days. After this, the lower hydrogel released MSCs to promote macrophage M2 polarization for anti-inflammation and induced MSCs osteogenic differentiation for fracture healing.

**Fig. 6:**
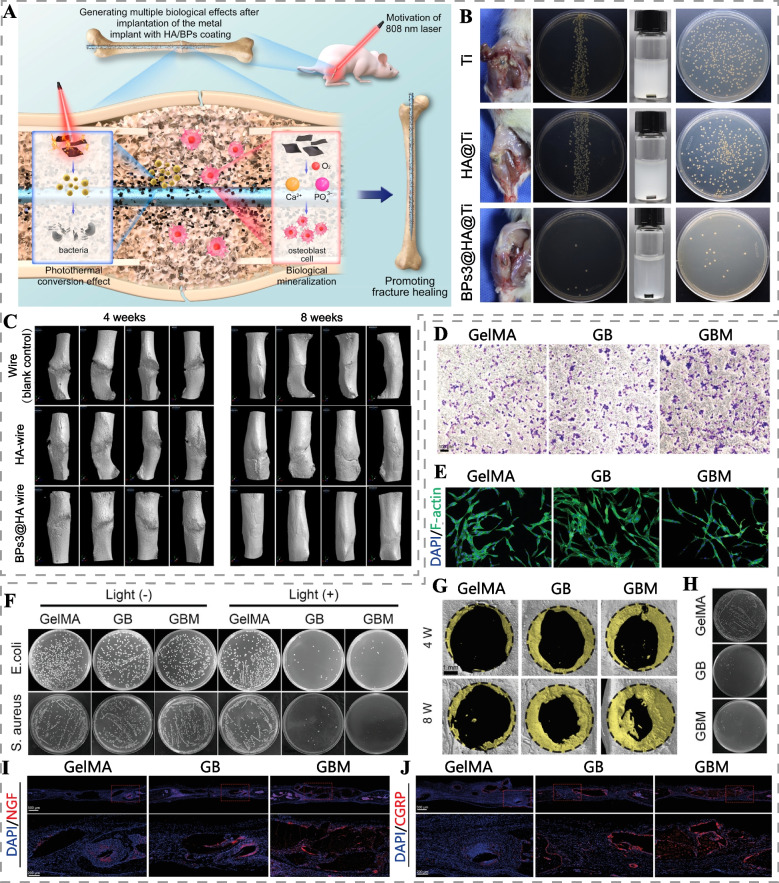
Photothermal bone scaffolds for the treatment of bone infections. **A** Schematic diagram of the mechanism of BPs@HA composite coating to promote fracture healing. **B** Representative photographs of the surgical site (the implant was inserted into the cancellous bone of the tibia just below the tibial plateau) and colonies of bacteria from the implant surface. **C** Micro-CT 3D reconstruction of femur fracture sites after treatment with wire (stainless Kirschner wire), HA-wire, and BPs@HA-wire. Reproduced with permission [73]. Copyright 2022, American Chemical Society. **D** Transwell migration assay of Schwann cells treated with GelMA, GB (GelMA loaded with BP), GBM hydrogel. Scale bar: 100 μm. **E** Immunostaining of PC12 cells cultured in Schwann cells conditioned medium on day 5 (GBM hydrogel effectively promoted neurite outgrowth). **F** Antibacterial capacity of different hydrogels with and without laser irradiation. Micro-CT 3D reconstruction images **G** and colony number **H** of *S. aureus*-infected skull defects treated with different hydrogels and periodic 808 nm (1 W/cm^2^) laser irradiation. **I** NGF and **J** CGRP immunofluorescence staining images of skull tissue. Scale bar: 500 μm (top), 200μm (bottom). Reproduced with permission [75]. Copyright 2023, Wiley-VCH

Photothermal materials exert broad-spectrum antibacterial effects through photothermal conversion under laser irradiation, raising the local temperature to induce bacterial membrane rupture, protein denaturation, and DNA damage [9]. While bacteria can develop mechanisms such as reducing uptake, inactivating, and promoting efflux of anti-microbial substances, they are unable to develop resistance to heat generated by PTT. Combining antibacterial PTT with pro-bone repair drugs represents an effective strategy for treating infected bone defects. For instance, Yuan et al. constructed a composite coating of BP and hydroxyapatite (BPs@HA) on the implant surface, which employed PTT at 50°C to eliminate bacteria, alleviate inflammation, and promote fracture healing through the bioactive coating (Fig. 6A-C) [73]. To prevent tissue damage from high temperature, Wu et al. proposed a "Sequential Photothermal Mediation" strategy for treating infected bone defects [74]. In this study, BP nanosheets with zinc sulfonate ligand (ZnL_2_) were integrated onto hydroxyapatite scaffolds (ZnL_2_-BPs@HAP). The PTT temperature was controlled at 47-50°C for the initial three days to eliminate bacteria and reduce inflammatory cell infiltration. Subsequently, the PTT temperature was lowered to 40-42°C to minimize tissue damage while accelerating bone regeneration through mild PTT and the release of Zn^2+^ and PO_4_^3-^.

### Combined therapy

Heat generated by PTT and ROS generated by PDT are the primary antibacterial pathways in phototherapy. However, it is essential to acknowledge that these therapeutic modalities can potentially be harmful to healthy tissues [13]. To mitigate these concerns and optimize treatment outcomes, researchers are exploring the development of multifunctional antibacterial bone implants that reduce reliance on monotherapy and minimize associated tissue damage. Currently, a range of studies is investigating the combination of PTT or PDT with chemotherapy and CDT, as well as incorporating physical structures to achieve synergistic antibacterial effects in the treatment of bone infections (Table 1).

**Table 1:** Phototherapy-based antibacterial bone scaffolds

Antibacterial strategy	Implant	Tested bacteria	Animal species	Research stages	Laser	Outcome	Ref.
PTT/PDT	TiO_2_/MoSe_2_/CHI-coated Ti implant	*Streptococcus mutans*	rat	in vitro, in vivo	808 nm, 0.6 W/cm^2^	Antibacterial; disrupted biofilm, and promoted implant osseointegration in *Streptococcus mutans* -infected tibia	[76]
	SPEEK-GO-BFP	*S. aureus, E. coli*	rabbit	in vitro, in vivo	808 nm, 0.5 W/cm^2^	PTT and PDT synergistically killed bacteria; promoted MC3T3 cells proliferation, migration, adhesion, and osteogenic differentiation, accelerated implant osseointegration at femoral defects	[77]
PTT/PDT/CDT	CuS@GP-CS/Fu ENFC	*S. aureus, E. coli*	-	in vitro	980 nm, 1.25 W/cm^2^	Antibacterial; released Cu^2+^ and fucoidan (Fu) to promote ALP expression and calcium nodule formation in 7F2 osteoblasts	[78]
PTT/chemotherapy	PLLA/ZIF-8@GO	*S. aureus, E. coli*	-	in vitro	808 nm, 1.0 W/cm^2^	Scaffolds synergistically antibacterial and disrupted biofilms via PTT and Zn^2+^; promoted proliferation of MG-63 osteoblasts	[79]
PTT/PDT/chemotherapy	CuS@BSA/rGO-PDA-coated Ti implant	*S. aureus, E. coli*	rat	in vitro, in vivo	808nm, 2.0 W/cm^2^	Resisted bacterial infection of bone tissue and disrupted biofilm, promoted vascularized bone regeneration	[80]
PTT/chemotherapy/physical puncture	TNS-MPN-AMP-coated Ti implant	*S. aureus, E. coli*	rat	in vitro, in vivo	808 nm, 0.5 W/cm^2^	Physical puncture, AMP release, and PTT synergistic anti-infection; promoted adhesion of MC3T3-E1 cells and formation of hydroxyapatite on the implant surface	[81]
PTT/PDT/chemotherapy/physical puncture	TiO_2_:FYH/Cur/BMP-2 nanorods	*S. aureus, E. coli*	mouse and rabbit	in vitro, in vivo	1060 nm, 0.6 W/cm^2^ in mouse, 1.0 W/cm^2^ in rabbit	Antibacterial, disrupted biofilm, inhibited inflammation, promoted proliferation, adhesion and differentiation of osteoblasts, accelerated implant osseointegration	[82]
PTT/PDT/electron transfer	GO/NCDs/Hap/Ti	*S. aureus, E. coli*	rat	in vitro, in vivo	808 nm, 0.5 W/cm^2^	Generation of photocurrents between the material and cells, leading to Ca^2+^ flow, accelerated migration, adhesion, and differentiation of MC3T3-E1 osteoblasts in vitro; electron transfer inhibited the adenosine triphosphate process of bacteria, synergized with PTT and PDT to eliminate tibial infections and inhibit inflammation; promoted angiogenesis	[83]

Combining PDT and PTT has emerged as a highly effective strategy for treating bone infections. In this approach, bone implants generate heat through photothermal conversion under laser irradiation, which inhibits bacterial viability and increases bacterial cell membrane permeability, enhancing their susceptibility to ROS [9]. This enables fewer ROS and lower temperatures to eliminate infections, resulting in powerful antibacterial effects and less tissue damage. Recently, Li et al. prepared BMP-2 microsphere-coated BPs@PLGA scaffolds (BMP-2@BPs) for PTT and PDT of infected bone defects [44]. In vivo, mild PTT at 41°C, in synergy with PDT, killed over 99% of methicillin-resistant *Staphylococcus aureus* (MRSA). Furthermore, the combination of mild PTT and BMP-2 effectively promoted the proliferation and osteogenic differentiation of periosteal-derived stem cells, accelerating the formation of new bone in infected cranial defects. Notably, infections often lead to localized nerve fiber necrosis, hindering the repair of infected bone defects [69, 75]. To address this, Jing et al. developed a photosensitive conductive GBM hydrogel, gelatin methacrylate (GelMA) hydrogel loaded with magnesium-modified black phosphorus [75]. GBM hydrogel promoted Schwann cell migration and neurite outgrowth of PC12 cells via NGF secreted by Schwann cells and the released Mg^2+^ from hydrogel (Fig. 6D, E). In addition, GBM hydrogel showed potent bactericidal effects against *Staphylococcus aureus* (*S. aureus*) and *Escherichia coli* (*E. coli*) via PTT and PDT (Fig. 6F). In vivo, GBM hydrogel effectively killed bacteria localized in infected skull defects under NIR irradiation, promoted NGF expression and the number of CGRP-positive nerve fibers (CGRP is a key mediator connecting nerve and bone regeneration), and accelerated the repair of *S. aureus*-infected skull defects (Fig. 6G-J).

Bacteria can exist in two forms: floating cells and microbial aggregates, with the latter known as biofilms, characterized by microbial aggregates embedded in extracellular polymeric substances composed of polysaccharides, proteins, extracellular DNA, and lipids [84]. Due to the physical barrier effect, biofilms exhibit enhanced resistance, up to 1000 times greater than planktonic bacteria, owing to biofilm heterogeneity, adaptive stress response, and the formation of persistent cells [85]. To disrupt biofilms, increasing the local temperature through PTT is a primary approach to increase penetration of antibacterial drugs. Yang et al. developed PLLA/ZIF-8@GO scaffolds that increased the local temperature to approximately 50°C through photothermal conversion, enhancing the penetration of the antibacterial agent Zn^2+^ and achieving highly efficient antibacterial properties [79]. Similarly, Yuan et al. constructed a composite coating containing mesoporous polydopamine (MPDA), indocyanine green (ICG), and arginylglycylaspartic acid (RGD) peptides on Ti surface to obtain Ti-M/I/RGD implant [86]. This innovative design facilitated the penetration of ICG into the biofilm and increased the permeability of bacterial cell membranes via PTT. Subsequently, ROS were generated via PDT, effectively disrupting biofilm integrity and eradicating bacteria.

## Bone tumors

Bone tumors encompass primary tumors and metastatic bone tumors [7]. Among them, osteosarcoma is the most common primary solid malignant bone tumor, traditionally treated with complete surgical resection of the tumor and chemotherapy. However, despite the introduction of chemotherapy, the long-term survival rates of 65%-70% remain unsatisfactory, and chemotherapy inevitably causes serious side effects such as nausea, hair loss, and bone marrow suppression [87]. Bone metastasis, a common complication of various cancers, is typically incurable and leads to pain and pathological fractures, significantly impacting patients' quality of life and survival [88]. Given the limitations of traditional treatment modalities, phototherapy has garnered increasing attention in the treatment of osteosarcoma and bone metastases due to its remote controllability, high spatial and temporal resolution, and minimally invasive nature [89, 90]. Tumor phototherapy shares a similar cell-killing mechanism with antibacterial phototherapy, relying on local thermotherapy generated by PTT and ROS generated by PDT to induce tumor cell death [91]. To achieve efficient and precise therapeutic effects, combining phototherapy with other treatments, such as chemotherapy and immunotherapy is a feasible strategy. The rapid advancements in nanomedicine, along with the current state of clinical treatment, have stimulated extensive research on light-responsive nanomaterials for bone tumor treatment. This section will introduce several new phototherapy strategies in the diagnosis and treatment of bone tumors.

### Light-responsive chemotherapeutic drug delivery and imaging systems

**Fig. 7:**
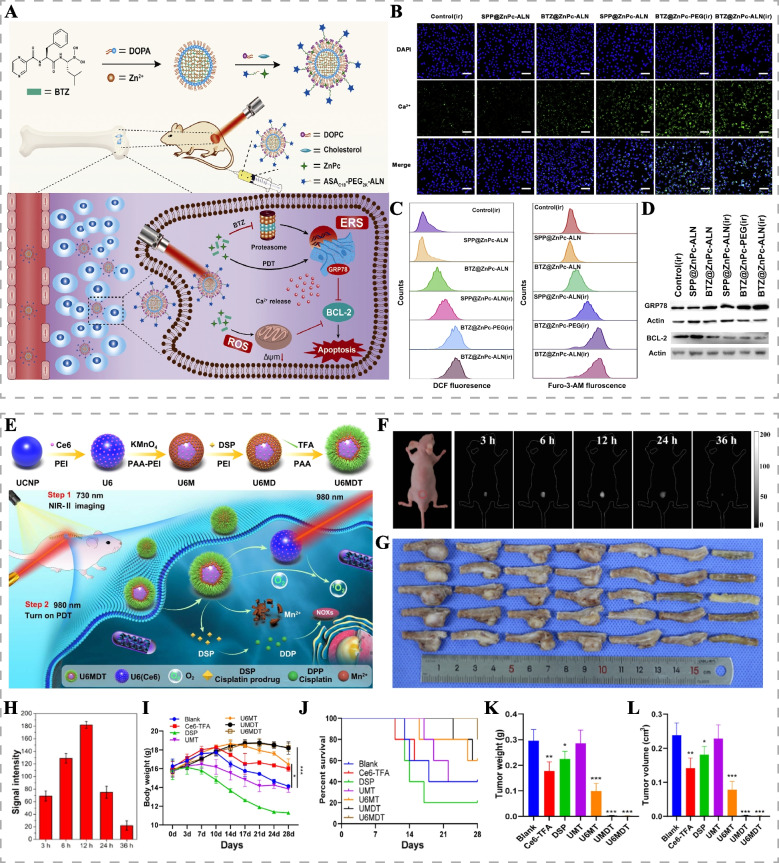
Light-responsive chemotherapeutic drug delivery and imaging systems for bone tumors. **A** Schematic diagram of the therapeutic mechanisms of BTZ@ZnPc-ALN for chemo-photodynamic therapy for bone metastatic breast cancer. **B** Cytosolic Ca^2+^ levels detection of MDA-MB-231 cells by confocal laser scanning microscopy using Fluo-3-AM as probe after different treatments. Scale bar: 50 μm. **C** Intracellular ROS production and cytosolic Ca^2+^ levels detection of MDA-MB-231 cells by flow cytometry. **D** Western blot analysis of ERS-related protein GRP78 and apoptosis-related protein BCL-2 after different treatments. Reproduced with permission [99]. Copyright 2020, Elsevier. **E** Schematic illustration of the synthetic and therapeutic mechanisms of UCNP/Ce6@mMnO2/DSP-TFA (U6MDT) for image-guided PDT-chemotherapy combination for the treatment of NSCLC-SM. **F** NIR fluorescence images and **H** signal intensity analysis of the NSCLC-SM mouse model at different times after intravenous injection of U6MDT. **G** Images of spinal vertebral tumors of each group in panel **I** after 4 weeks of treatment. **I** Body weight, **J** survival curve, **K** tumor weight and **L** tumor volume of NSCLC-SM model in different treatment groups (*p < 0.05, **p < 0.01 and ***p < 0.001). Reproduced with permission [94]. Copyright 2022, American Chemical Society

Light-responsive nanomaterials with excellent photothermal conversion properties and photocatalytic properties have been extensively studied [92–94]. However, single phototherapy alone often falls short of achieving satisfactory therapeutic outcomes. To address this limitation, drug delivery systems mediated by light-responsive nanomaterials have been developed, enabling precise and efficient delivery of anticancer drugs while harnessing excellent photodynamic and photothermal therapeutic effects. This synergistic approach significantly improves the anti-tumor efficacy, showing great promise in treating chemotherapy-resistant bone tumors [95]. By employing strategies like surface modification and the addition of targeting ligands, functionalized light-responsive drug delivery systems demonstrate high drug delivery rates, extended blood circulation time, increased bone tumor accumulation, and reduced side effects [96, 97]. Sun et al. developed a dopamine and bone-targeting ligand-modified light-responsive drug delivery system (BTZ/Fe^2+^@BTF/ALD) loaded with the CDT catalyst (Fe^2+^) and anticancer drug bortezomib (BTZ) for targeted PTT/chemotherapy/CDT for breast cancer bone metastasis [92]. BTZ/Fe^2+^@BTF/ALD exhibited remarkable photothermal conversion efficiency and bone-targeting ability, accumulating at the site of bone metastasis and releasing BTZ and Fe^2+^ in response to the acidic and hydrogen peroxide (H_2_O_2_)-rich tumor microenvironment. This led to effective tumor growth inhibition. Additionally, it has been reported that BTZ, a proteasome inhibitor, not only promotes the accumulation of ubiquitinated proteins in the endoplasmic reticulum (ER) and induces endoplasmic reticulum stress (ERS) by inhibiting the clearance process of misfolded proteins but also enhances intracellular ROS generation [98]. Capitalizing on this finding, Huang et al. prepared an alendronate (ALN)-modified nanoagent (BTZ@ZnPc-ALN) encapsulating BTZ and the photosensitizer Zinc phthalocyanine (ZnPc) for chemo-photodynamic therapy of breast cancer bone metastasis (Fig. 7A) [99]. BTZ@ZnPc-ALN exhibited prolonged blood circulation time, strong bone affinity, and pH-responsive drug release. The combination of PDT and BTZ aggravated intracellular oxidative stress, induced mitochondrial damage, and further intensified ERS, synergically inducing tumor cell death (Fig. 7B-D).

The development of a light-responsive chemotherapeutic drug delivery system capable of visualizing bone tumor tissues holds promising clinical significance. Such a system can provide structural information and spatial tumor location, guiding treatment decisions and enabling real-time monitoring of the treatment process, enabling precise treatment and improving patient prognosis. Liu et al. developed multifunctional nanoparticles (PDA-ALN/DOX) composed of polydopamine (PDA), doxorubicin (DOX), and ALN [97]. PDA displayed good photothermal conversion and drug-carrying capacity. ALN facilitated bone targeting and carried Fe ions to enhance the T1-weighted image signal intensity, enabling synergistic PTT and chemotherapy of osteosarcoma under the guidance of magnetic resonance imaging (MRI). Yang et al. developed multifunctional hydrogels (HG-CAHs) integrating PTT, NIR/pH-responsive DOX release, CT imaging, and fluorescence imaging for the treatment of osteosarcoma [100]. Gold nanoclusters endowed the hydrogels with excellent fluorescence and CT imaging capabilities, enabling real-time monitoring of HG-CAHs enrichment in osteosarcoma and guiding the combination therapy. Han et al. prepared lanthanum-ion-doped upconversion nanoparticles (UCNPs)-based nanomaterials (UCNP/Ce6@mMnO_2_/DSP-TFA) (Fig. 7E) [94]. UCNPs activated the photosensitizer Ce6 to exert PDT by achieving upconversion luminescence under 980 nm laser irradiation, while 730 nm laser excited downconversion luminescence of UCNPs for NIR-II imaging. UCNP/Ce6@mMnO2/DSP-TFA could release cisplatin drug (DSP) in response to the tumor microenvironment to synergize with chemotherapy and PDT for the treatment of spinal metastasis of non-small cell lung cancer (NSCLC-SM) (Fig. 7F-L).

### Combination therapy with immunotherapy

Phototherapy, while showing good antitumor effects, faces challenges in completely eradicating tumors due to distant metastasis and tumor recurrence [101]. Immunotherapy, on the other hand, has received widespread attention for its potential to stimulate the host immune system to attack and destroy tumor cells. However, current clinical reports show that only a small percentage of patients benefit from immunotherapy, which may be attributed to the low immunogenicity of tumors and the tumor’s immunosuppressive microenvironment [102]. To overcome these challenges, there is an urgent need to develop new therapeutic strategies.

Phototherapy has the potential to enhance immunotherapeutic efficacy by inducing immunogenic cell death (ICD). Dying tumor cells after phototherapy release damage-associated molecular patterns (DAMPs) and tumor-associated antigens, promoting dendritic cell maturation, T cell activation, and cytotoxic T lymphocyte infiltration, leading to a robust immune response [103, 104]. Consequently, the combination of phototherapy and immunotherapy, known as photoimmunotherapy, emerges as a promising approach to overcome the limitations of monotherapy and maximize their respective advantages. This approach not only targets the primary tumor but also addresses distantly metastatic tumor cells, offering hope to patients with distant metastasis and recurrence.

**Fig. 8:**
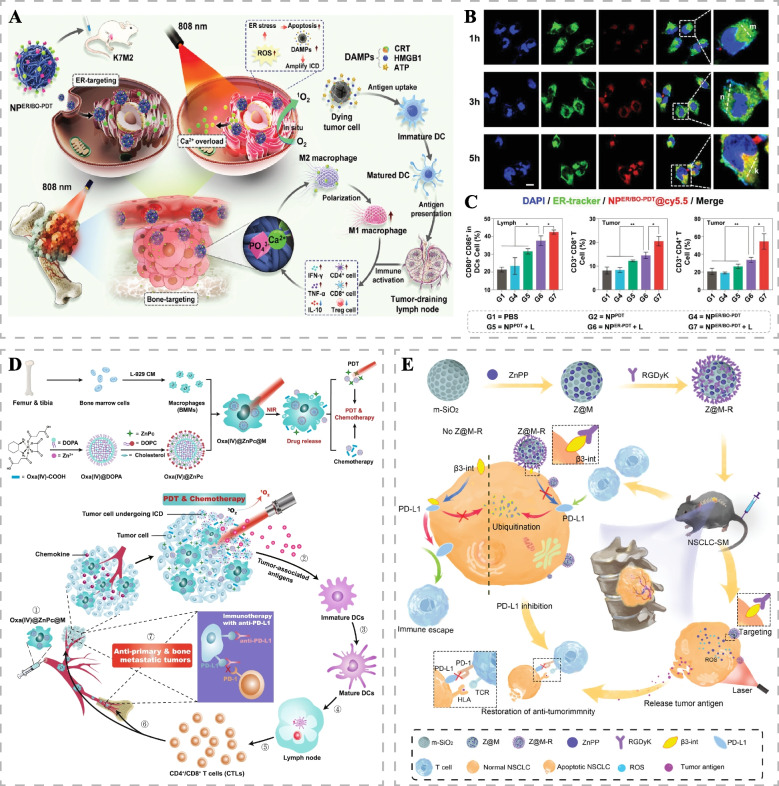
Combination therapy with immunotherapy for bone tumors. **A** Schematic diagram of the therapeutic mechanisms of NP^ER/BO-PDT^. **B** ER targeting of NP^ER/BO-PDT^ in MNNG/HOS cells at different times. Scale bar: 10 μm. **C** The percentages of mature DCs in tumor-draining lymph nodes and percentages of CD8 T cells and CD4 T cells in tumor tissues of K7M2 tumor-bearing mice after different treatments (*p < 0.05 and **p < 0.01). Reproduced with permission [105]. Copyright 2022, Wiley-VCH. **D** Schematic illustration of the synthetic and therapeutic mechanisms of Oxa(IV)@ZnPc@M for chemo-photodynamic therapy of primary and bone metastatic breast cancer. Reproduced with permission [106]. Copyright 2021, Springer Nature. **E** Schematic diagram of the synthetic and therapeutic mechanisms of ZnPP@MSN-RGDyK for NSCLC-SM. Reproduced with permission [107]. Copyright 2022, Wiley-VCH

ER is considered a potential target for inducing ICD and generating a strong immune response [103]. Zhang et al. developed cascade targeting nanoparticles (NP^ER/BO-PDT^), capable of highly accumulating and exerting PDT in bone tumors and ER (Fig. 8A, B). This design overcomes the challenges posed by aberrant proliferation of tumor cells and the complex anatomical aberrations of tumors, which often limit drug delivery efficiency [105]. Under NIR irradiation, ROS generated by NP^ER/BO-PDT^ effectively killed tumor cells and induced sustained ERS, leading to the release of DAMPs from tumor cells and amplifying ICD. This robust immune response in tumor-bearing mice enabled highly efficient photodynamic immunotherapy (Fig. 8C). While the immunostimulatory properties of phototherapy can enhance antitumor immunity, ICD induced by PTT or PDT alone might produce a weak and transient immune response insufficient to effectively halt tumor progression. Huang et al. developed engineered macrophages (Oxa(IV)@ZnPc@M) carrying the photosensitizer ZnPc and chemotherapy drug oxaliplatin prodrug (Oxa(IV)) [106]. Under NIR, ZnPc and Oxa(IV) were released, effectively killing primary breast cancer and enhancing ICD induction through the combination of PDT and chemotherapy (Fig. 8D). This promoted the intratumoral infiltration of antitumor lymphocytes, effectively combating primary and bone metastatic breast cancer. Furthermore, macrophages overcame biological barriers to accurately deliver ICD inducers and polarized to the M1 phenotype to enhance anti-tumor immune responses. With the addition of programmed cell death ligand 1 (PD-L1) blockade, Oxa(IV)@ZnPc@M demonstrated stronger anti-bone metastasis ability with long-term immune memory effects.

Programmed cell death protein 1 (PD-1) and PD-L1 are essential immune checkpoint molecules, and overexpression of PD-L1 in cancer cells inhibits the function of effector T cells, leading to immune evasion. Anti-PD-1/PD-L1 monoclonal antibodies have been extensively studied and approved for use in various tumors, but their therapeutic efficacy remains suboptimal [108, 109]. Compared with immune checkpoint inhibitors that directly block PD-1/PD-L1 interactions, mesoporous silicon nanoparticles (ZnPP@MSN-RGDyK) designed by Zhou et al. showed excellent inhibition of the occurrence and progression of NSCLC-SM through the synergistic immunotherapeutic effects of integrin *β*3 (*β*3-int)-specific inhibitor (RGDyK) and Zinc protoporphyrin (ZnPP) [107]. RGDyK downregulated PD-L1 expression by targeting *β*3-int, promoting PD-L1 ubiquitination, while ZnPP-mediated PDT-generated ROS, thus promoting the release of tumor antigens (Fig. 8E). This subsequently increased tumor-infiltrating lymphocytes and reversed the immunosuppressive microenvironment. ZnPP@MSN-RGDyK demonstrated therapeutic potential in NSCLC-SM patients susceptible to resistance to PD-1 inhibitors.

### Bifunctional Biomaterials

The invasion of bone tumors and surgical intervention often lead to extensive bone defects, further complicated by factors such as insufficient blood supply and potential bacterial infections, making the repair of bone defects challenging [110]. Tumor recurrence caused by residual tumor cells after surgery significantly impacts patient prognosis. Therefore, there is a clinical demand for nanomaterials with dual functions: killing tumor cells and promoting bone regeneration.

**Fig. 9:**
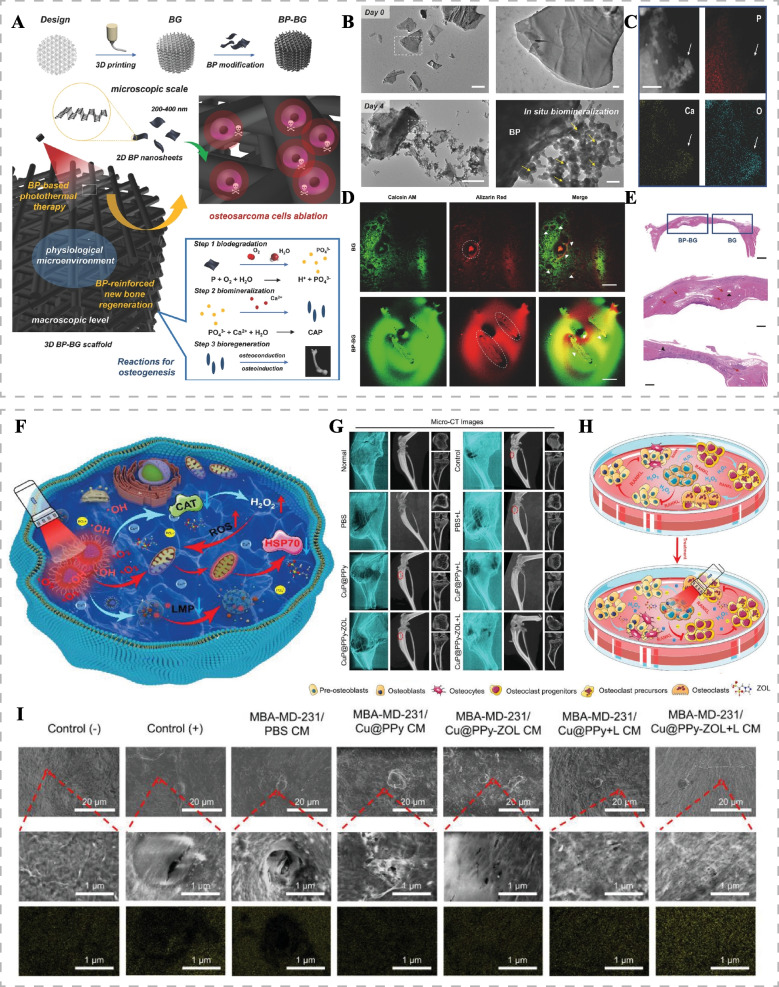
Bifunctional Biomaterials for bone tumors. **A** Schematic illustration of the synthetic and therapeutic mechanisms of BP-BG scaffolds for the stepwise treatment of osteosarcoma. **B** Transmission electron microscopy images of BP nanosheets dispersed in simulated body fluids on days 0 and 4. Scale bar: 0.5 μm (left), 50 nm (right). **C** The high-angle annular dark field image and P, Ca, and O elemental mappings after 4 d of biodegradation. Scale bar: 50 nm. **D** Confocal fluorescence images (scale bar, 200 μm) and **E** H&E staining (top, scale bar 1 mm; middle and bottom, scale bar, 400 μm) of newborn bone tissue at the site of cranial defects in Sprague-Dawley rats implanted with BG and BP-BG scaffolds at week 6. Reproduced with permission [111]. Copyright 2018, Wiley-VCH. **F** Schematic diagram of the anti-tumor mechanism of CuP@PPy-ZOL NPs via synergistic PDT/PTT/CDT. **G** 3D micro-CT reconstruction images of metastatic tumor tibia at different angles in a mouse model of breast cancer bone metastasis after different treatments. **H** Schematic diagram of the interaction mechanism of MDA-MB-231 cells with RAW264.7 cells and MC3T3-E1 cells before and after treatment. **I** Scanning electron microscope images and energy dispersive X-ray elemental mapping of calcium on the cranial surface of RAW264.7 cells cultured with skull bone for 7 days after different treatments. Reproduced with permission [116]. Copyright 2023, Wiley-VCH

Integrating the tumor-killing effect of phototherapy with the osteogenic activity of bioactive substances presents a promising strategy for repairing bone defects and preventing tumor recurrence after bone tumor surgery. Yang et al. developed bioglass scaffolds (BP-BG scaffolds) incorporating black phosphorus nanosheets for the stepwise treatment of osteosarcoma [111]. The black phosphorus nanosheets first applied PTT to kill tumor cells at the postoperative defect site. Subsequently, they degraded, releasing PO4^3-^, which induced in situ biomineralization, guiding bone regeneration by extracting calcium (Fig. 9A-C). BP-BG scaffolds facilitated osteoblast adherence, proliferation, and differentiation, ultimately leading to the formation of new bone tissues on the progressively degraded scaffolds (Fig. 9D, E). Other ions, such as Ca, Mg, Si, Fe, Sr, and Cu ions, have also been reported to promote osteogenic differentiation or angiogenesis. Light-responsive nanomaterials containing these ions can effectively repair bone defects while exerting phototherapeutic effects to kill tumor tissues [112–114]. Xu et al. developed calcium phosphate composites (TCP-FePSe_3_) doped with FePSe_3_ nanosheets to inhibit the postoperative recurrence of osteosarcoma and promote bone regeneration [115]. FePSe_3_ nanosheets exerted PTT and released Se ions to activate the caspase-dependent apoptosis pathway, inducing anti-tumor effects. TCP-FePSe_3_ promoted vascularized bone regeneration by releasing bioactive Fe, P, and Ca ions, thereby achieving bone repair.

Tumor metastasis to bone disrupts the tightly regulated balance between osteoclasts and osteoblasts, leading to osteoclast activation and inhibition of bone matrix production by osteoblasts, causing bone erosion and bone defects [117]. Restoring the balance between bone resorption and bone regeneration can be achieved by inhibiting osteoclast activity, thereby inhibiting bone metastasis and osteolysis [118]. Zeng et al. developed NIR-II responsive nanoplatforms (CuP@PPy-ZOL NPs) to synergize with PTT/PDT/CDT to kill tumors, inhibit osteoclast activity, and disrupt the "vicious cycle" of osteolytic bone destruction, remodeling the bone microenvironment [116]. Under 1064 nm laser irradiation, mild PTT of CuP@PPy-ZOL NPs, together with released Cu^2+^, PO4^3-^ and zoledronic acid (ZOL), promoted osteogenic differentiation and bone repair, thus inhibiting osteolytic breast cancer bone metastasis (Fig. 9F-I). ZOL, known to preferentially enrich in sites with high osteoclast activity, inhibits osteoclast-mediated bone resorption and is the standard treatment for patients with bone metastasis to reduce the risk of skeletal-related events. Sun et al. developed bone-targeting nanoparticles (Au@MSNs-ZOL) by coupling gold nanorods encapsulated in mesoporous silica nanoparticles with ZOL, effectively treating breast cancer bone metastasis [119]. Au@MSNs-ZOL induced tumor cell apoptosis via PTT under NIR irradiation, inhibited osteoclast differentiation, promoted osteoblast differentiation, and alleviated bone resorption and bone pain.

## Osteoarthritis

Osteoarthritis is a degenerative joint disease characterized by the destruction and loss of cartilage in the joints, leading to joint pain, swelling, stiffness, and impaired mobility [120]. Approximately 300 million people worldwide are affected by OA, and this number is expected to rise as the population ages and the prevalence of obesity increases [120, 121]. Currently, common medications used for OA treatment, such as nonsteroidal anti-inflammatory drugs (NSAIDs) and glucocorticosteroids, primarily aim to alleviate symptoms rather than cure OA and may lead to gastrointestinal and renal adverse effects with prolonged use [122]. Therefore, the search for novel, effective, and highly targeted therapies with minimal side effects to prevent OA progression is crucial. Recently, NIR-responsive drug delivery systems have emerged as promising solutions with advantages such as precise and controllable drug delivery, improved drug stability, prolonged drug residence time, and reduced need for repeated intra-articular drug injections, making them highly promising for OA treatment [123].

### Phototherapy strategies for cartilage protection and cartilage regeneration

In progressive OA, cartilage undergoes aberrant metabolic changes characterized by increased chondrocyte apoptosis, reduced synthesis of collagen II and proteoglycans, and enhanced breakdown of cartilage matrix driven by matrix metalloproteinase (MMP). These changes are strongly associated with inflammation, oxidative stress, mitochondrial dysfunction, abnormal mechanical stimuli, and other physicochemical factors [124]. Protecting chondrocytes and restoring the balance between cartilage matrix synthesis and breakdown are essential therapeutic strategies for OA. Recently, Xu et al. constructed NIR-responsive epigallocatechin gallate (EGCG)-modified Au-Ag nanoplatforms [125]. The nanoplatforms utilized the peroxidase (POD) activity in combination with NIR-triggered release of the anti-inflammatory agent EGCG to reduce chondrocyte apoptosis induced by H_2_O_2_. This approach reversed the trend of decreased type II collagen expression and increased MMP expression, effectively protecting cartilage from damage in OA rats in vivo.

**Fig. 10:**
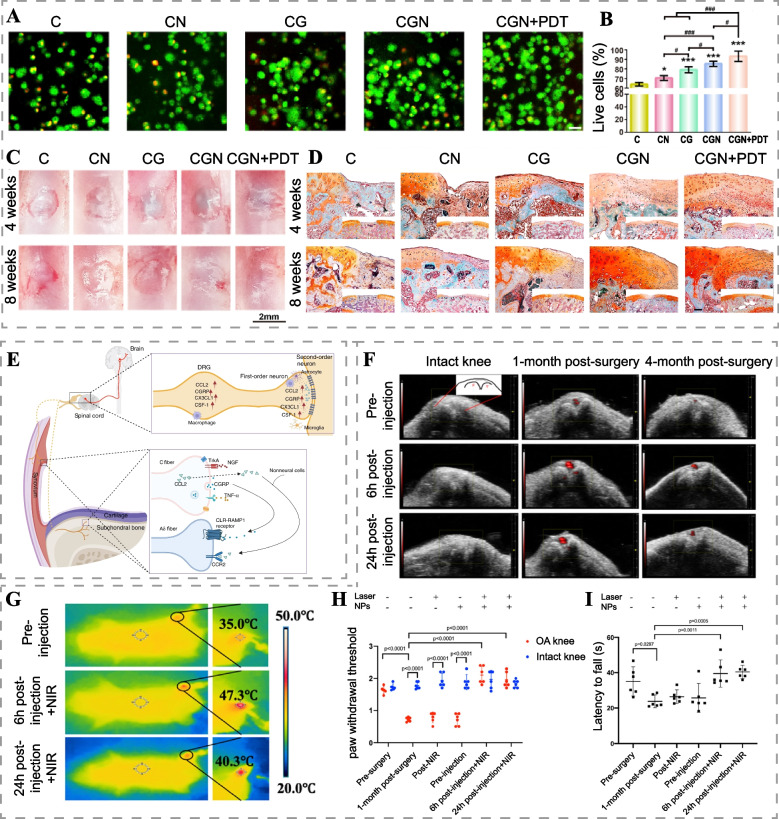
Phototherapy techniques for OA treatment. **A** Live/dead assay images of BMSCs cultured in scaffolds with or without PDT on day 21. Scale bar: 20 μm. **B** Statistical analysis of live/dead assay of BMSCs (*, ^#^ indicate *p <* 0.05, ^##^ indicates *p <* 0.01, ***, ^###^ indicates *p <* 0.001). **C** Gross images and **D** Safranin-O/fast green staining images of cartilage defects after 4 and 8 weeks of treatment. Scale bar: 20 μm. Reproduced with permission [130]. Copyright 2019, Elsevier. **E** OA pain-related signaling pathways. Reproduced with permission [132]. Copyright 2022, Springer Nature. **F** US-PA overlay images of intact knee and surgical DMM-induced posttraumatic OA mouse knee (1 month postoperative and 4 months postoperative) at different time points after intravenous injection of Anti-NGF-MoS_2_-AuNR. **G** Infrared thermography of OA mice (1 month postoperative) after intravenous injection of anti-NGF-MoS_2_-AuNR. **H** Paw withdrawal threshold and **I** motor coordination of mice 1 month postoperative. Reproduced with permission [133]. Copyright 2021, American Chemical Society

Cartilage defects are an important pathologic feature of OA, and articular cartilage repair is challenging due to the lack of blood vessels, nerves, and lymphatics in the cartilage tissue [126]. MSCs have shown promise for cartilage repair due to their accessibility, self-renewal, and pluripotent nature [127]. Appropriate levels of ROS have been reported to play a role in promoting stem cell growth and chondrogenic differentiation [128, 129]. Recently, Lu et al. constructed collagen-genipin-carbon dots nanocomposite hydrogels loaded with BMSCs (CGN hydrogels) [130]. Incorporating carbon dot nanoparticles (CD NPs) in the hydrogel increased its stiffness by 21-fold, activating the TGF-β/Smad2/3 signaling pathway in BMSCs. Under 808 nm laser irradiation, the CGN hydrogel generated ROS through PDT and activated the mTOR signaling pathway in BMSCs. The synergistic effects of the enhanced hydrogel stiffness and ROS generated by PDT promoted proliferation and chondrogenic differentiation of BMSCs, leading to accelerated cartilage tissue regeneration (Fig. 10A-C). And the new cartilage tissue fused well with the original cartilage, offering hope for long-lasting cartilage repair (Fig. 10D). Although ROS can regulate the proliferation and differentiation of MSCs, however, related studies have found that the viability of MSCs decreases after too high power irradiation, which may be related to excessive ROS production [130, 131]. Hence, further exploration of the optimal ROS level and mechanism for inducing BMSCs chondrogenic differentiation will be essential for future advancements in this field.

### Phototherapy strategies for regulating joint inflammation

In OA, cartilage destruction and synovial inflammation create a vicious circle where cartilage degradation products activate inflammatory cells, leading to the release of inflammatory factors in large quantities. In turn, these inflammatory signals disrupt chondrocyte metabolism, inhibit cartilage synthesis, and accelerate cartilage breakdown [124]. Therefore, targeting joint inflammation is crucial for slowing the progression of OA. Zhao et al. developed a chitosan-modified molybdenum disulfide platform loaded with dexamethasone (MoS_2_@CS@Dex) [134]. After injection into the joints, MoS_2_@CS@Dex stayed for more than 48h and released dexamethasone (Dex) upon NIR stimulation. This inhibited the release of TNF-α and IL-1β from activated macrophages, alleviating joint inflammation and protecting cartilage tissue. Similarly, Chen et al. designed a novel nitrogen monoxide (NO) nanogenerator NO-Hb@siRNA@PLGA-PEG (NHsPP) for Notch1-siRNA delivery and promoting near-infrared photothermal conversion-promoted NO gas delivery [135]. Consequently, this activated the AMPK pathway and down-regulated the Notch pathway, inhibiting the release of pro-inflammatory factors from macrophages and mitigating inflammation-induced cartilage erosion and degeneration.

Conventional therapeutic oligonucleotides have limitations in terms of instability and cellular uptake. In a recent study, antisense DNA sequences of IL-1β mRNA were modified onto gold nanorods to create spherical nucleic acids (SNAs) [136]. By modifying hyaluronic acid (HA) with DNA complementary to the SNA sequences, DNA-grafted (^DNA^HA) was obtained. HA-SNAs were then formed by base-pairing ^DNA^HA with SNAs. HA-SNAs remained in mouse knee joints for more than one month after injection. Under NIR-promoted photothermal conversion, DNA hydrogen bonds in HA-SNAs were broken, releasing SNAs to interfere with IL-1β mRNA molecules, which further down-regulated MMP-1 and MMP-13 whilst up-regulating synthetic components (Col2α, aggregated glycans) in chondrocytes, thus effectively protecting cartilage tissues.

### Osteoarthritis analgesia

Chronic pain has long been the predominant symptom of OA, yet the source of its pain is unknown. Joint cartilage lacks nerve innervation, so the synovium and subchondral bone are considered the primary sources of joint pain because they are densely innervated by sensory and sympathetic nerve [137]. Nociceptor nerve terminals in joints express a variety of ion channels that are activated in response to chemical (e.g. nerve growth factor, TNF-α and IL-1β), mechanical or thermal harmful stimuli, resulting in pain signaling (Fig. 10E) [132]. Subsequently, the expressions of chemokine (C-C motif) ligand 2 (CCL2), calcitonin gene-related peptide (CGRP), NLRP3 and Wnt/β-catenin in nociceptor cells bodies were up-regulated [132, 138]. Eventually, secondary neurons within the dorsal horn of the spinal cord are activated by neurotransmitters (glutamate, CGRP, and substance P) and transmit signals to the higher central nervous system to produce nociception. In addition to injurious pain mechanisms, pain in patients with OA involves peripheral and central sensitization [137]. Upon activation of nociceptors, upregulated CGRP and CCL2 are released from activated C-fiber terminals and bind to adjacent Aδ nerve fibers' calcitonin receptor-like receptor (CLR) and receptor activity-modifying protein 1 (RAMP1), or chemokine (C-C motif) receptor 2 (CCR2), leading to nerve sensitization, resulting in abnormal mechanical pain and hyperalgesia in the periarticular region of OA patients [138].

Given that pain is a prominent symptom of OA, accurate assessment and effective analgesia are essential to improve the quality of life of patients with OA [139]. Clinically, often a substantial discrepancy between OA pain symptoms and imaging findings can be noted [140]. Therefore, OA pain assessment relies on subjective patient descriptions and semi-quantification of visual analog scores. Recently, Au et al. designed an anti-NGF antibody-coupled MoS_2_ nanosheet-coated gold nanorod (anti-NGF-MoS_2_-AuNR) for the evaluation and treatment of OA pain [133]. By targeting the OA pain-causing factor nerve growth factor (NGF), anti-NGF-MoS_2_-AuNR remained in the OA knee joint after intravenous injection, allowing PAI of NGF-targeted pain for visualization and quantification of pain (Fig. 10F). Furthermore, the nanoplatform achieved joint pain relief for up to 7 days by blocking the binding of NGF to its receptor TrkA and using NIR-promoted localized thermal therapy on the OA knee, as evidenced by improved paw withdrawal threshold and motor coordination in OA mice (Fig. 10G-I). This extended duration of analgesia outperformed the 24-hour efficacy of NSAIDs. Although antagonizing NGF relieves OA pain, recent studies have suggested that this may exacerbate joint damage [141]. Therefore, in subsequent studies, attention should also be paid to whether there is consistency in analgesia and relief of OA progression.

While phototherapy has primarily been used to build NIR-responsive drug delivery platforms for modulating inflammation and protecting chondrocytes in OA, it is crucial to note that most of these platforms currently require joint injection, which may increase the risk of joint infections [125]. The lack of vascularization in the joint cavity also limits the entry of antibacterial drugs into the joint, making it challenging to address joint infections [142]. Therefore, exploring PTT and PDT that specifically target OA joints for joint antibacterial therapy and OA treatments holds great significance for enhancing patient outcomes and safety. Further research and development in this direction will pave the way for more effective and comprehensive treatments for OA.

## Rheumatoid Arthritis

Rheumatoid arthritis (RA) is a chronic progressive autoimmune disease characterized by inflammation of the synovium, pannus formation, and progressive destruction of cartilage and bone [143]. RA affects approximately 1% of the world's population and presents with symmetrical pain and swelling in multiple joints, often affecting small joints in the hands and feet, as well as large joints like knees and shoulders [143, 144]. If left untreated, RA can cause joint deformities, leading to disability, reduced quality of life, and significant healthcare costs [145]. While disease-modifying antirheumatic drugs (DMARDs) (methotrexate, sulfonamides, TNF inhibitors, or inhibitors of Janus kinase) and glucocorticoids are commonly used to control RA progression, and NSAIDs for pain relief, more than 20% of patients experience poor treatment outcomes, in addition to adverse effects associated with the drugs used in the treatment of RA [144, 146].

The pathogenesis of RA remains to be thoroughly elucidated, but genetic and environmental factors, including microbial infections, smoking, and obesity, are thought to play important roles in its development [147]. In RA joints, aberrant autoimmunity activates macrophages, which further activate fibroblast-like synoviocytes (FLS) by releasing pro-inflammatory cytokines such as TNF-α, IL-1β, and IL-6. Macrophages and FLS accumulate in large numbers in the synovium, triggering infiltration of mast cells, neutrophils, and lymphocytes via chemokines, exacerbating synovial inflammation and leading to pannus formation [148]. Pannus invades the periarticular bone, causing erosion of bone and cartilage through the release of MMP and induction of osteoclast formation [147, 148].

Conventional organic photosensitizers (photofrin or haematoporphyrin derivatives, benzoporphyrin derivatives) have been used since the 1990s for the destruction of pathologic synovial tissue in RA joints [149]. However, conventional organic photosensitizers have limitations such as skin phototoxicity, poor solubility, and limited tissue penetration of excitation light, which thwart their clinical translation [150]. Encapsulation or modification of photosensitizers can improve their solubility, stability, and selectivity, enhancing their effectiveness for PDT in RA treatment [151, 152]. For example, Gallardo-Villagrán liganded the photosensitizer tetrapyridylporphyrins (TPyP) to dinuclear arene ruthenium(ii) complexes to enhance TPyP solubility for PDT of RA [151]. In another study, Gabriel et al. designed a photosensitizer activated by high levels of thrombin in the RA joints by linking the photosensitizer unit through a thrombin-cleavable peptide, thereby improving its selectivity [153]. In recent years, significant progress has been made in the development of inorganic light-responsive nanomaterials for the treatment of RA. These nanomaterials, including pure metals, metal-organic frameworks, and carbon-based nanomaterials, offer several advantages, such as high ROS productivity, efficient photothermal conversion efficiency, and the ability to tailor their structures and compositions [154]. Leveraging these properties, researchers have successfully constructed light-responsive platforms for targeted and effective phototherapy in RA. Therefore, we herein summarize the advances in the therapeutic strategies and applications of phototherapy in RA.

### Phototherapy for FLS and activated macrophage clearance

Currently, the most prominent phototherapy strategy for treating RA involves utilizing PTT or PDT to target and eliminate pathogenic cells in the affected joints, thereby reducing the expression of inflammatory factors and interfering with synovial hyperplasia, as well as bone and cartilage destruction [155, 156]. However, the non-specific nature of heat and ROS generated by PTT and PDT poses a challenge in achieving targeted therapy. Therefore, researchers have actively sought avenues to maximize the selectivity of phototherapy in RA joints.

One effective strategy involves the enhanced permeability and retention (EPR) effect, which utilizes nanoparticles of specific sizes to cross immature blood vessels in RA tissue and accumulate within the joint [157]. For instance, Dorst et al. constructed liposomes loaded with the photosensitizer IRDye700DX, designed to accumulate in the joints via the EPR effect and phagocytosed by macrophages, thus improving the photosensitizer’s selectivity in vivo [158]. The choice of particle size is crucial for effective targeting, as sizes exceeding endothelial cell gaps cannot penetrate blood vessels, while smaller particles may rapidly diffuse between tissues and blood vessels, leading to reduced retention time [159]. Studies have shown that particle sizes ranging from 100 nm to 220 nm offer optimal RA joint targeting [157, 159].

Another promising approach involves utilizing light-responsive nanomaterials encapsulated with cell membranes from erythrocytes, macrophages, FLS, or hybridized membranes, which can actively target RA tissues through ligand-receptor binding mechanisms. These modified nanomaterials not only enhance their stability in the bloodstream but also exhibit excellent biocompatibility and immunogenicity [152, 160]. Furthermore, recent research has revealed specific molecular expressions in key effector cells activated in RA joints, such as high CD44 expression in FLS and macrophages [160, 161]. Leveraging the antigen-antibody or ligand-receptor specific binding principles, modified photosensitizers and light-responsive nanoplatforms can actively target and eliminate RA key effector cells, thus improving the selectivity of PTT and PDT in RA treatment at both tissue and cellular levels [162]. Herein, we summarize representative studies that have employed PTT or PDT to target RA effector cells, providing valuable insights into the advancement of targeted phototherapy for RA (Table 2).

**Table 2:** Targeted phototherapy based on molecular expression characterization of RA effector cells

Cell targeting	Targets/cell marker	Modification	Conjugation	Laser	Research stages	Animal species	Outcomes	Ref.
FLS	Vasoactive intestinal peptide (VIP) receptor	VIP	VIP-ATO-GPx/CuS@HA	808 nm, 0.5 W/cm^2^	in vitro, in vivo	CIA mouse	Inhibited FLS energy metabolism and synergized with PTT and CDT to induce FLS apoptosis, achieving inhibition of synovial inflammation and bone and cartilage destruction	[163]
	CD44	HA	HA@RFM@PB@SE NPs	808 nm, 1 W/cm^2^	in vitro, in vivo	AIA rat	Chemotherapy and PTT synergistically inhibited FLS proliferation and alleviated joint inflammation	[160]
	fibroblast activation protein (FAP)	Anti-FAP antibody 28H1	28H1-700DX	690 nm, 17.6 or 52 J/cm^2^	in vitro, in vivo	CIA mouse	Targeted FAP-positive FLS and induced cell death via PDT to alleviate joint inflammation	[162]
Activated Macrophages	Folate receptor β	Folate	FAGMs	808 nm, 1 W/cm^2^	in vitro, in vivo	AIA rat	Chemotherapy synergized with PTT to eradicate activated macrophages, attenuate joint inflammatory cell infiltration and cartilage damage, and reduce serum inflammatory factor levels	[164]
	Folate receptor β	Methotrexate (MTX)	Au-DEN-MTX-IR780 NP	808 nm, 0.5 W/cm^2^	in vitro	-	Chemotherapy and PTT synergistically induced ROS generation and activated macrophages death	[165]
	CD44	HA	Pd@Se-HA NPs	808 nm, 0.5 W/cm^2^	in vitro, in vivo	CIA mouse	Suppressed macrophage infiltration, ROS production, and cytokine-mediated joint inflammation	[161]
	Scavenger receptor	Dextran sulfate (DS)	QRu-PLGA-RES-DS NPs	808 nm, 0.4 W/cm^2^	in vitro, in vivo	CIA mouse	PAI for RA diagnosis, photothermal modulation of resveratrol (RES) release, increased M2/M1 macrophage ratio, and alleviation of joint inflammation	[166]
endothelial cell	Rvβ3 integrin	RGD	Pd-Cys@MTX@RGD nanosheets	808 nm, 0.3 W/cm^2^	in vitro, in vivo	CIA mouse	PAI for RA diagnosis, PTT and MTX synergistically inhibited the inflammatory response induced by VEGF and IL-1β, and protected articular cartilage	[167]

*Abbreviations*: *CIA* collagen-induced arthritis, *AIA* adjuvant-induced arthritis

In healthy joints, FLS produces lubricin and hyaluronic acid to maintain synovial fluid homeostasis [168]. In contrast, in RA joints, FLS accumulates in large numbers in the vascular opacities and exhibits an aggressive phenotype with direct release of MMP mediating cartilage erosion and activation of nuclear factor kappa-β ligand (RANKL) receptor, inducing osteoclast formation and bone erosion [169].

**Fig. 11:**
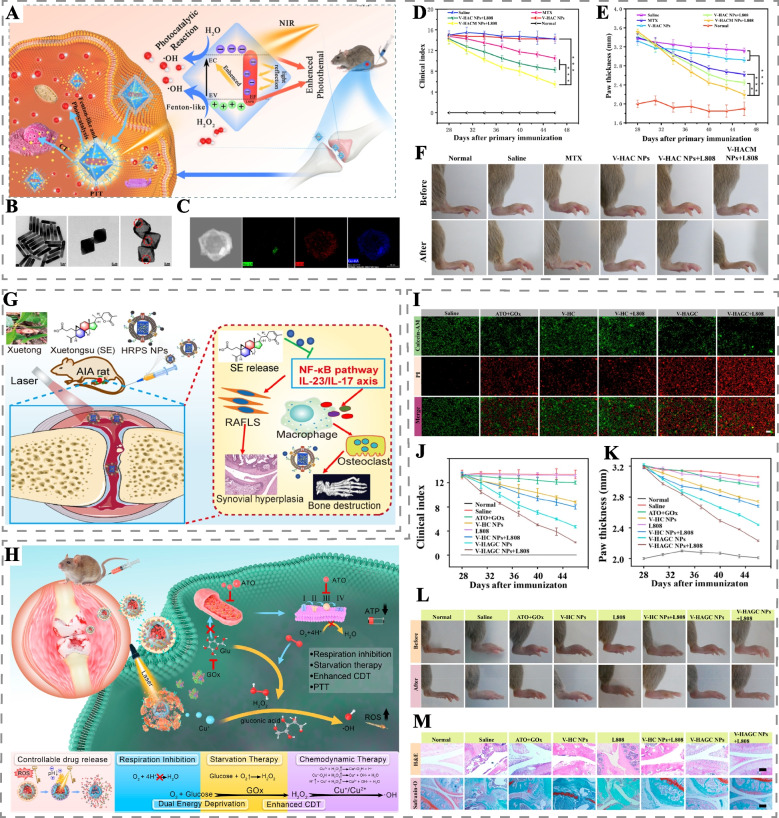
Phototherapy techniques for RA effector cells clearance. **A** Schematic illustration of VIP-HA-Au NR@CuS NPs for the treatment of RA. **B** Transmission electron microscope images of Au NR (left), Au NR@CuO NPs (center), Au NR@CuS NPs (right). Scale bar: 20 nm (left), 50nm (middle), 50nm (right). **C** EDS elemental mapping images of Au NR@CuS NPs. **D** Clinical index of RA and **E** paw thickness of RA mice after treatment at different time points. *p < 0.01, ***p < 0.001. **F** Joint morphology of RA mice before and after treatment. Reproduced with permission [170]. Copyright 2021, Elsevier. **G** Schematic illustration of the mechanism of HRPS NPs in the treatment of RA. Reproduced with permission [160]. Copyright 2022, American Chemical Society. **H** Schematic diagram of V-HAGC NPs for the treatment of RA by energy inhibition/CDT/PTT. **I** Live/dead staining images of FLS after different treatments. Scale bar: 100 μm. **L** Joint morphology and **M** H&E and Safranin-O staining images of RA mice treated with PBS, ATO + GOx, V-HC NPs and V-HAGC NPs with or without 808 nm irradiation. Scale bar: 100 μm. Reproduced with permission [163]. Copyright 2022, American Chemical Society

Over the past three years, PTT and PDT-targeted killing of FLS has proven effective in interfering with RA progression in animal models, making it a highly promising therapeutic strategy for RA [170]. Ha et al. constructed MTX-loaded multifunctional nanoparticles, and under NIR irradiation, the combination of 0.13 μM MTX with PTT exhibited a stronger FLS killing compared to 30 μM MTX alone [171]. This reduction in drug dosage helps avoid potential side effects. Huang et al. developed vasoactive intestinal peptide (VIP) and hyaluronic acid-modified Au NR@CuS yolk-shell NPs (VIP-HA-Au NR@CuS NPs) for targeting FLS (Fig. 11A) [170]. The unique yolk-shell structure allowed for multiple light reflection capabilities, resulting in a photothermal conversion efficiency of up to 67% (Fig. 11B, C). The synergistic killing of FLS by PTT, PDT, chemotherapy using MTX, and Fenton-like reaction of copper synergistically inhibited RA progression (Fig. 11D-F). In addition, Yu et al. encapsulated the anti-RA drug schisandra chinensis lactone E (SE) with Prussian blue nanoparticles, further encapsulated with a biomimetic membrane and modified with HA to obtain HRPS NPs [160]. These HRPS NPs targeted FLS, killing about 70% of them through the chemotherapeutic effect of SE and PTT. They also reduced FLS-mediated inflammation by inhibiting the IL-23/IL-17 axis and the NF-κB pathway (Fig. 11G). The oxidative stress state of FLS plays a key role in the hyperinflammatory state and chondrocyte destruction in RA. Antioxidant therapy has been shown to alleviate joint inflammation and treat RA effectively [172]. The rapid proliferation of FLS has been reported to be highly dependent on glycolysis [173]. Therefore, Qiu et al. proposed a dual energy suppression strategy based on respiratory inhibition and starvation therapy to combat RA (Fig. 11H) [163]. Their constructed VIP-ATO-Gox/CuS@HA (V-HAGC) NPs targeted FLS to conserve O_2_ by inhibiting the mitochondrial oxidative phosphorylation system (OXPHOS) aerobic metabolism via atovaquone (ATO). Subsequently, glucose oxidase (Gox) in the nanoparticles consumed glucose and O_2_ to produce H_2_O_2_, providing enough substrate for the Fenton reaction to produce •OH. The synergized PTT and enhanced CDT effectively killed more than 80% of FLS and alleviated cartilage and bone destruction in RA mice (Fig. 11I-M).

In RA joints, circulating monocytes migrate to synovial membrane under the action of chemokines and transform into pro-inflammatory macrophages, exacerbating synovial inflammation. Under the stimulation of RANKL from FLS and osteoblasts, they can even differentiate into osteoclasts, directly participating in bone erosion [174]. Depletion of these macrophages is undesirable, as they are the first responders of the immune system. Instead, researchers are exploring targeted nanoplatforms based on the antigenic expression characteristics of activated macrophages to selectively eliminate them through combination therapy [164, 175]. Recently, Li et al. developed PEG-FA functionalized semiconducting polymer quantum dots (SP) hybrid mesoporous silica nanoparticles (SMPFs) targeting macrophages via folate (FA) [176]. The SMPFs depleted local oxygen via PDT and activated the hypoxia-activated prodrug tirapazamine, resulting in the synergistic elimination of macrophages and alleviation of RA progression through PTT, PDT, and chemotherapy. Additionally, ameliorating macrophage oxidative stress and reversing the pro-inflammatory phenotype of macrophages can alleviate local inflammation in RA [174]. Zheng et al. constructed Pd@Se-HA NPs that target macrophages, and through ROS scavenging and PTT, they synergistically inhibited the expression of multiple pro-inflammatory factors in macrophages, preventing joint degeneration [161]. RA joints are hypoxic due to a highly dysfunctional microvascular system and over-infiltrated heterogeneous cells [177]. Reversing the localized hypoxic microenvironment in inflammatory joints reduces ROS accumulation and alleviates joint inflammation, proving to be an effective strategy for RA treatment [178]. One study encapsulated oxygen-saturated perfluoro-*n*-pentane (PFP) by biodegradable poly(dl-lactide-co-glycolic acid) phase-transition nanoparticles aiming to oxygenate the joints and improve the hypoxic microenvironment using NIR-responsive mode [179]. Wang et al. constructed polyethylene glycol-modified cerium dioxide shell-coated gold nanorods (Au@CeO_2_), which scavenge ROS and oxygenate by catalyzing H_2_O_2_ to produce oxygen. This improved the joint hypoxic microenvironment and down-regulated the expression of hypoxia-inducible factor-1 (HIF-1) in the joints. PTT, on the other hand, ablated inflammatory cells, alleviating inflammation and strengthening the catalytic oxygen-producing ability of CeO_2_. In vivo, this combined PTT, ROS scavenging, and oxygen therapy effectively suppressed synovial inflammation and cartilage destruction, demonstrating excellent RA treatment outcomes [178].

Overall, direct delivery of oxygen is limited in its ability to relieve joint hypoxia. Instead, the nanozyme-based oxygen production through H_2_O_2_ consumption shows promise in simultaneously reversing oxidative stress and hypoxic microenvironments, as well as increase the catalytic rate under photothermal modulation, presenting a more promising modality for the treatment of RA [180].

### Light-responsive diagnostic and treatment platform

**Fig. 12:**
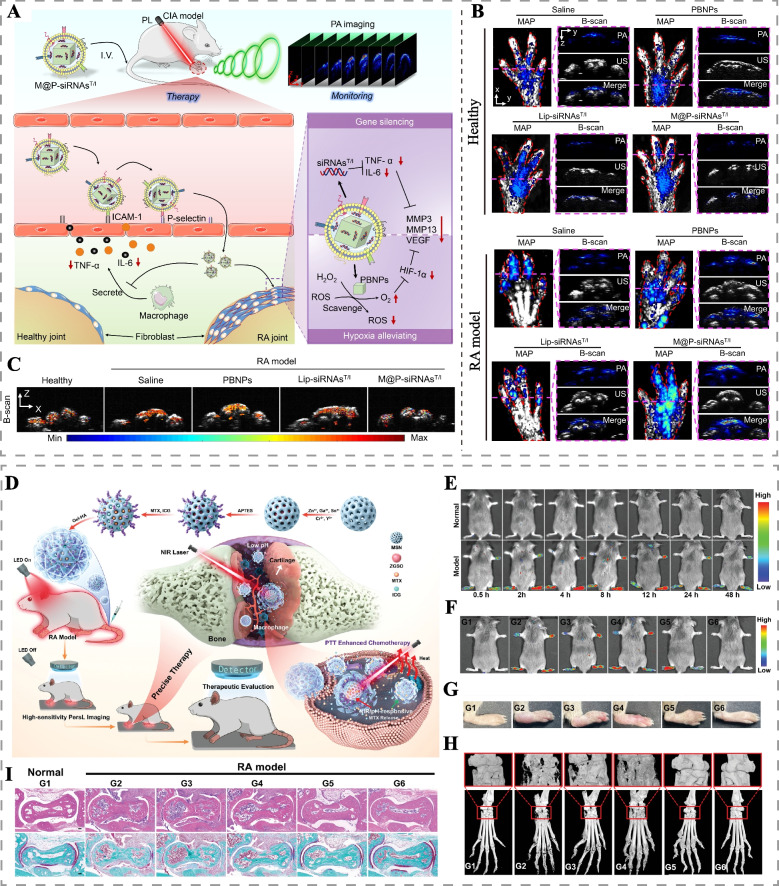
Light-responsive diagnostic and therapeutic integrated platform for RA treatment and monitoring. **A** Schematic illustration of M@P-siRNAsT/I for PAI-guided RA therapy. **B** PAI of healthy and RA mice treated with saline, PBNPs, Lip-siRNAs^T/I^ and M@P-siRNAs^T/I^ for detecting the ability to target RA joint (MAP, maximum amplitude projection; B-scan, cross section image). **C** Forepaw PAI (B-scan) after 49 days of treatment to assess the degree of hypoxia (PA signals obtained at 725 nm and 800 nm wavelengths were used to construct oxygen saturation maps, rainbow shows the relative PA blood oxygen signal). Reproduced with permission [185]. Copyright 2022, PNAS. **D** Schematic diagram of the construction process of mZMI@HA nanoprobes and their high-sensitivity PersL imaging-guided RA therapy. **E** PersL images of healthy and RA mice after treated mZMI@HA at different time points. **F** PersL images of mice after complete treatment to assess efficacy. **G** Joint morphology and **H** micro-CT 3D reconstruction images of mice after treatment. **I** H&E (top) and Safranin-O (bottom) staining of ankle joints after treatment. (G1: normal mice, G2: RA model of PBS, G3: RA model of PBS with laser, G4: RA model of ICG with laser, G5: RA model of MTX, G6: RA model of mZMI@HA with laser.) Reproduced with permission [188]. Copyright 2023, Wiley-VCH

Early diagnosis of RA is crucial to treat the joint before it becomes irreversible. MRI and ultrasound (US) imaging are used for early inflammation detection in the joints. However, MRI is time-consuming, costly, and potentially contraindicated, while US imaging is dependent on the experience of the operator [181]. PAI has gained attention as a non-invasive, low-cost, high-contrast, real-time continuous imaging technique for early diagnosis of RA [182]. PAI utilizes light absorption by hemoglobin and the difference in the absorption spectra of oxygenated and deoxygenated hemoglobin to detect local neovascularization, blood flow, and hypoxia in joints without the need for exogenous imaging agents [183, 184]. For RA diagnosis, integrated nanoplatforms using exogenous contrast agents have been developed. Recently, TNF-α and IL-6 silencing siRNA (siRNA^T/I^) and Prussian blue nanoparticles (PBNPs) encapsulated by macrophage membrane vesicles (MMVs) have been used to develop biomimetic nanoparticles M@P-siRNAs^T/I^ which target RA joints (Fig. 12A) [185]. PBNPs allowed PAI to assess joint targeting of M@P-siRNAs^T/I^ and also mimicked peroxidase, catalase, and superoxide dismutase activities, scavenging of ROS and production of oxygen, which synergized with the inhibitory effects of siRNA^T/I^ on inflammatory factor expression, inhibited joint inflammation and alleviated hypoxia, as well as ameliorated joint erosion (Fig. 12B, C). In another study, nanoparticles consisting of dextran sulfate (DS)-modified thermosensitive molecular poly (lactic-*co*-glycolic acid) (PLGA) as a shell and quadrilateral ruthenium (QRu) as a core, QRu-PLGA-RES-DS NPs, with photothermal and PAI capabilities were developed [166]. PAI of QRu provided information on the in vivo distribution of nanoparticles, and photothermal functional modulation of resveratrol (RES) release increased the M2/M1 macrophage ratio, enabling PAI-guided RA therapy. NIR-II PA imaging has higher spatial resolution and deeper tissue penetration (i.e., more than 10 cm) compared to NIR-I PA imaging [186]. Recently, Chen et al. constructed the first NIR-II PAI nanoprobes utilizing tocilizumab (TCZ)-coupled polymeric nanoparticles (TCZ-PNPs) for the diagnosis and treatment of RA [187]. A signal-to-noise ratio of up to 35.8 dB has been reported for TCZ-PNPs, allowing them to monitor the RA condition by displaying swelling in cartilage tissues. After one month of treatment with TCZ-PNPs, the progression of RA was observed to be inhibited in mice.

NIR persistent luminescence nanoparticles (NIR-PLNPs) continuously emit NIR persistent luminescence (PersL) after stopping excitation light irradiation [189]. Recently, Wang et al. constructed mZMI@HA nanoprobes targeting activated macrophages for guided therapy and therapeutic evaluation in RA (Fig. 12D) [188]. After mZMI@HA was injected into the body, PersL imaging showed distinct signals in the paws of RA mice, suggesting that mZMI@HA accumulated at the site of RA onset (Fig. 12E). To scavenge activated macrophages and inhibit synovial proliferation and cartilage destruction, mZMI@HA also released MTX via NIR and pH-responsive forms. After complete treatment, PersL imaging with mZMI@HA was again administered intravenously, and low luminescent signal intensity was found in the paws of RA mice, which were consistent with joint morphology and histology, suggesting that PersL imaging with mZMI@HA can also be used for treatment monitoring and efficacy assessment (Fig. 12F-I). Fluorescence imaging is also used for in vivo distribution detection of nanomaterials. Recently, Pan et al. constructed Pt-MOF@Au@QDs/PDA for hydrothermal therapy targeting FLS, emitting fluorescent signals under 640 nm light excitation to determine joint distribution [190]. Further, Pt-MOF@Au acted as a visible light-activated H_2_ generator to produce H_2_ to scavenge •OH in FLS to alleviate cellular oxidative stress. Under NIR irradiation, Pt-MOF@Au@QDs/PDA induced FLS death via PTT. In vivo, H_2_ therapy synergized with PTT to alleviate joint inflammation and inhibit synovial proliferation as well as bone and cartilage erosion in RA mice.

## Skeletal muscle injury

Skeletal muscles constitute approximately 30-40% of the body's weight and are necessary for voluntary movement [191]. Minor injuries to skeletal muscle, such as strains and contusions, can be repaired through the proliferation and differentiation of muscle satellite cells, the stem cell population of skeletal muscle [192]. However, severe muscle injuries may lead to fibrosis, scarring, and loss of muscle function [193].

Heat therapy shows significant potential in the treatment of muscle injuries by alleviating muscle pain, promoting satellite cell proliferation, and reducing collagen fiber formation [194, 195]. Recently, Zhang et al. recently developed a microneedle patch (CW-PGE_2_-MN) containing carbonized wormwood and prostaglandin E_2_ (PGE_2_) for damaged muscle repair [196]. NIR-induced photothermal therapy for 20 minutes three times daily, combined with PGE_2_, promoted the proliferation and differentiation of muscle stem cells. After three weeks of treatment, muscle strength and myogenic fiber counts improved in muscle-damaged mice, indicating effective muscle repair.

It is essential to note that the muscle repair effect in this study was achieved through heat transfer from the skin patch to the deeper tissues. The skin temperature reached as high as 60°C after 5 minutes of NIR (808 nm, 3 W/cm^2^) irradiation, which may be harmful to the skin. Currently, research on photothermal therapy for muscle injuries is scarce, but previous studies have suggested that heat therapy at 40°C may promote muscle repair [196]. In the future, the construction of photothermal materials with high photothermal conversion efficiency capable of accurately targeting injured muscles for localized photothermal therapy will improve therapeutic efficacy and reduce damage to other tissues [197].

## Conclusion and outlook

In recent years, nanotechnology has advanced the development of multifunctional nanoplatforms based on PTT and PDT, showing great promise in addressing medical challenges related to musculoskeletal disorders. This article outlines the progress in strategies and applications of PTT and PDT for treating MSDs, considering the characteristics of different diseases. Specifically, for MSDs involving tissue damage, phototherapy directly modulates several key processes in tissue repair, such as promoting angiogenesis, modulating immune homeostasis, and promoting stem cell differentiation, thereby accelerating tissue repair. Meanwhile, synergistic therapies based on PTT and PDT have demonstrated a powerful clearance effect on bacteria and biofilms, which is essential for creating a microenvironment suitable for tissue regeneration. For bone tumors and RA, the intravenously administered light-responsive nanoplatforms can be accurately localized to the corresponding pathological tissues based on the EPR effect, modified targeting ligands. Therefore, phototherapy can be performed on the tumor or the affected joint without invasive procedures on the patient, which helps reduce the pain. In addition, for bone tumors, the construction of a multifunctional phototherapy nanoplatform can combine PTT and PDT with chemotherapy, CDT, gene therapy, immunotherapy, or starvation therapy to achieve precise and efficient tumor cell killing and even overcome tumor drug resistance. As for joint diseases, the light-responsive drug delivery nanoplatform can not only relieve pain symptoms through hyperthermia, but also extend the retention time of the loaded drug in the joint, and achieve light-responsive active drug delivery to delay disease progression.

Although phototherapy, as a non-invasive, highly selective, and controllable technique, has demonstrated significant therapeutic potential in MSDs, it is still subject to various limitations in the treatment of MSDs. First, the musculoskeletal system is often covered by skin and subcutaneous tissues, and there is inevitable energy scattering and absorption as light propagates through these tissues, which may reduce the efficacy of phototherapy. Secondly, for certain MSDs involving tissue damage, such as bone defects, the repair process is dynamic and complex, and phototherapy's regulation of cellular functions may not align well with this process. Furthermore, in the case of bone infections, bacteria can easily hide within the complex anatomical structures of the bone, leading to a risk of infection recurrence even after phototherapy. Moreover, the uneven distribution of photothermal agents and photosensitizers in tumors may lead to suboptimal tumor clearance. It's worth noting that bone fracture sites, tumor tissues, as well as joints affected by OA and RA often exhibit hypoxia, which could limit the effectiveness of highly oxygen-dependent PDT. Therefore, before phototherapy can be widely applied in the clinical treatment of MSDs, several challenges need to be addressed:

(i) Choice of light source: Most light sources currently used for the treatment of MSDs focus on NIR-I, although they have been shown to be effective in activating phototherapy in animals. In humans, however, the thickness of the skin and subcutaneous tissue is much greater than in animal models, which may hinder phototherapy. Currently, NIR-II lasers with deeper tissue penetration, lower tissue light absorption and scattering, and higher maximum permissible exposure (MPE) (1 W/cm^2^ at 1064 nm versus 0.33 W/cm^2^ at 808 nm) are believed to help reduce adverse tissue reactions and improve treatment outcomes. NIR-II-based imaging has superior imaging depth, spatiotemporal resolution, and signal-to-noise ratio, which helps to build more accurate diagnostic systems. In the future, studies need to further investigate the interaction between light and tissue to guide the selection of light for MSDs phototherapy.
(ii) Disease microenvironment: Developing phototherapy nanoplatforms must consider the disease microenvironment, such as low pH, hypoxia, high ROS, high GSH or specific enzyme expression. Considering the limitations of hypoxia in PDT, constructing nanoplatforms for oxygen supply or oxygen self-generation, combined with PTT to enhance blood circulation for improved oxygen supply, can enhance the efficacy of PDT. In addition, the design of multiple stimulus-responsive drug delivery systems with light- and microenvironment-responsiveness and various combination therapies can help to achieve on-demand drug delivery, increase drug utilization efficiency, improve the disease microenvironment and enhance therapeutic efficacy.
(iii) Biosafety: Ensuring the biosafety of phototherapy in MSDs treatment is of paramount importance. Phototherapy toxicity can arise from the light absorption by skin tissue, heat from PTT and ROS from PDT. Selecting light sources with larger wavelengths, improving photothermal conversion efficiency, shortening the irradiation time, controlling temperature and ROS production, and constructing targeted nanomaterials through ligand modification will help overcome these challenges. Further, long-term biotoxicity and metabolic pathways of nanomaterials in vivo need to be systematically studied prior to clinical translation.
(iv) Molecular and cellular mechanisms: Phototherapy has been demonstrated to modulate cellular functions, such as PTT promoting the osteogenic differentiation of MSCs and PDT promoting the chondrogenic differentiation of MSCs. Although some signaling pathways have been shown to be involved through traditional methods, researchers still need to utilize cutting-edge technologies such as single-cell transcriptomics, proteomics, and metabolomics to uncover more molecular regulatory mechanisms by which phototherapy influences cellular functions. In addition, MSDs often involve complex cellular mechanisms. For bone defect repair, current phototherapy strategies overemphasize anti-inflammation and ignore the benefits of appropriate levels of inflammation early in bone injury (e.g., removal of pathogens and damaged tissues). In the case of RA and OA, the complexity and incomplete understanding of pathogenesis make this a challenge for the application of phototherapy techniques. Therefore, emphasizing interdisciplinary collaboration and in-depth basic research to deepen the understanding of the molecular mechanisms of MSDs and phototherapy on cellular function will be key to advancing the development of new therapeutic strategies for MSDs.

In conclusion, phototherapy technology holds exciting potential for the treatment of MSDs. Continued research and optimization of phototherapy nanoplatforms towards versatility, precision, and efficiency for the treatment of bone, cartilage, and bone tumor diseases, along with exploring other MSDs therapies, will ultimately pave the way for clinical translation and benefit patients.

## Data Availability

Not applicable.

## Abbreviations

MSDs: Musculoskeletal disorders
RA: Rheumatoid arthritis
PTT: Photothermal therapy
PDT: Photodynamic therapy
ROS: Reactive oxygen species
CDT: Chemodynamic therapy
PAI: Photoacoustic imaging
NIR: Near-infrared
OA: Osteoarthritis
MSCs: Mesenchymal stem cells
HSP: Heat shock protein
Runx-2: Runt-related transcription Factor-2
ALP: Alkaline phosphatase
BMP: Bone morphogenetic protein
Col-I: Collagen I
PDGF: Platelet-derived growth factor
TGF: Transforming growth factor
VEGF: Vascular endothelial growth factor
BP: Black phosphorus
PLGA: Poly (lactic acid-glycolic acid copolymer)
SMPs: Shape memory polymers
3D: Three-dimensional
4D: Four-dimensional
CT: Computed tomography
RGD: Arginylglycylaspartic acid
ER: Endoplasmic reticulum
ERS: Endoplasmic reticulum stress
ALN: Alendronate
PDA: Polydopamine
MRI: Magnetic resonance imaging
NSCLC-SM: Spinal metastasis of non-small cell lung cancer
ICD: Immunogenic cell death
DAMPs: Damage-associated molecular patterns
PD-1: Programmed cell death protein 1
PD-L1: Programmed cell death ligand 1
ZOL: Zoledronic acid
MMP: Matrix metalloproteinase
NGF: Nerve growth factor
FLS: Fibroblast-like synoviocytes
EPR: Enhanced permeability and retention
HA: Hyaluronic acid
RANKL: Nuclear factor kappa-β ligand
VIP: Vasoactive intestinal peptide
FA: Folate
US: Ultrasound
